# Maturation stage-specific V-ATPase disassembly explains the neutral pH of mature mucocyst lysosome-related organelles in *Tetrahymena thermophila*

**DOI:** 10.1242/jcs.264146

**Published:** 2025-12-09

**Authors:** Ajay Pradhan, Niraj Tadasare, Debolina Sarkar, Vandna Maurya, Lavan K. Bansal, Aaron P. Turkewitz, Santosh Kumar

**Affiliations:** ^1^ National Centre for Cell Science, NCCS Complex, Savitribai Phule Pune University Campus, Ganeshkhind Road, Pune 411007, Maharashtra, India; ^2^ Regional Centre for Biotechnology, NCR Biotech Science Cluster, Faridabad 121001, Haryana, India; ^3^ Department of Molecular Genetics and Cell Biology, The University of Chicago, Chicago, IL 60637, USA

**Keywords:** LRO, Mucocyst, V-ATPase-a1p, V-ATPase, pHluorin, pH

## Abstract

Lysosome-related organelles (LROs) are a heterogeneous family of organelles found in many cell types, whose similarities to lysosomes include acidification by vacuolar-type proton ATPases (V-ATPases). However, some organelles with hallmarks of LROs are nonetheless non-acidic. Here, we investigate this phenomenon using the ciliate *Tetrahymena thermophila*, which has secretory LROs called mucocysts. Using three approaches, we show that mature mucocysts, poised for exocytosis, are non-acidic. However, mucocysts forming in the cytoplasm are acidic, and a specific V-ATPase a-subunit is present and indispensable for mucocyst biogenesis. In the absence of this subunit, cells show defects in at least two features of mucocyst formation, namely heterotypic vesicle fusion of mucocyst precursors and proprotein processing. The stage specificity of acidification can be explained by our finding that several other canonical V-ATPase subunits are present in the forming mucocysts but not in mature mucocysts. Based on our data, we argue that a specific V-ATPase complex is targeted to newly forming, immature mucocysts and subsequently disassembles at a later stage in the maturation pathway.

## Teaser

**Summary:** We show that the V-ATPase a1-subunit is essential for lysosome-related organelle (LRO) biogenesis in *Tetrahymena*. A specific V-ATPase complex acidifies new LROs then disassembles, resulting in a neutral-pH mature compartment.

## INTRODUCTION

Lysosome-related organelles (LROs), which include mammalian Weibel–Palade bodies, melanosomes, acrosomes, basophil granules, lamellar bodies and platelet-dense granules, are a family of organelles whose morphological and functional diversity belies the fact that they commonly depend on a set of highly conserved proteins for their biosynthesis (Delevoye et al., 2019; Khawar et al., 2019; Le et al., 2021; Marks et al., 2013; McCormack et al., 2017; Metcalf et al., 2008; Weaver et al., 2002). Though sharing features with both late endosomes and lysosomes, LROs are functionally, morphologically and/or compositionally distinct (Ambrosio and Di Pietro, 2025; Luzio et al., 2014; Marks et al., 2013; Montero-Hernández et al., 2025). They are formed by a multistep process in which an immature organelle matures by acquiring cargo from both secretory pathways as well as traffic from endosomes (Bonifacino, 2004; Dell'Angelica et al., 2000; Raposo et al., 2007). Many of the proteins in LROs rely on receptors and cytoplasmic adaptors for their targeting as well as complexes to tether and fuse vesicles (Banushi and Simpson, 2022; Marks et al., 2013). These include the AP-3 adaptor, the homotypic fusion and protein sorting (HOPS) complex, the small GTPases Rab32 and Rab38, the sortilin/VPS10 sorting receptors, and three biogenesis of LROs (BLOC) complexes (Banushi and Simpson, 2022; Bultema et al., 2012; Delahaye et al., 2014; Gautam et al., 2006; Hermey, 2009; Kaur et al., 2017; Starcevic et al., 2002; Wasmeier et al., 2006; Yong et al., 2025). Phylogenetic analysis of LRO machinery has revealed that these LRO components were present in the last eukaryotic common ancestor (LECA) and constitute core elements of eukaryotic membrane trafficking (More et al., 2024).

One constituent broadly shared among LROs is the vacuolar-type proton ATPase (V-ATPase) complex, whose presence drives acidification of the LRO lumen. Acidification plays a crucial role for many types of LROs (Dell'Angelica et al., 2000; Goff et al., 2025; Matsumoto and Nakanishi-Matsui, 2019; Pejler et al., 2017; Sun-Wada et al., 2003). For instance, T lymphocytes and natural killer cells synthesize LROs called lytic granules, which maintain an acidic pH essential for the targeted release of macromolecules to eliminate virally infected cells or tumor cells (Griffiths and Argon, 1995). Type II alveolar epithelial cells synthesize lamellar bodies that store lung surfactant. The processing, and therefore release, of surfactant proteins from lamellar bodies requires an acidic pH (Chander et al., 1986; Chintagari et al., 2010; Weaver et al., 2002).

V-ATPases are composed of two macromolecular domains, V_1_ and V_0_. V_1_, the catalytic domain, is a 650 kDa peripheral complex comprising A_3_B_3_CDE_3_FG_3_H subunits that hydrolyzes ATP to generate the energy required for pumping protons. V_0_ is a 260 kDa integral membrane protein complex that forms a proton channel; it consists of ac_8_c′c′′def subunits (Breton and Brown, 2013; Forgac, 2007; Futai et al., 2019). Thus, both the V_1_ and V_0_ subunits are required to form a fully functional holo-V-ATPase complex. Since V-ATPases are active in diverse intracellular compartments, compartment-specific stimulation of assembly or disassembly might be one organizing principle of the endomembrane network. For example, progressively increasing V-ATPase assembly might account for increased acidity following endocytosis and transport through endosomes in mammalian cells (Lafourcade et al., 2008). Conversely, the enzymatic activity of the complex can be downregulated by disassembly, in which the V_1_ domain separates from the membrane-integral V_0_ domain (Oot et al., 2017; Parra et al., 2014). In yeast and mammalian cells, reversible V-ATPase assembly and disassembly have been reported in response to stimuli including glucose and amino acid starvation, refeeding and oxidative stress (Bodzęta et al., 2017; Chan and Parra, 2014; Khan et al., 2022; Sautin et al., 2005; Stransky and Forgac, 2015).

In addition to compartment-specific assembly and/or disassembly, V-ATPase activities can be tailored for different compartments based on the presence of paralogs, historically called isoforms, for some subunits (Toei et al., 2010). In yeast, two a-subunit isoforms are encoded by *VPH1* and *STV1* (Manolson et al., 1992, 1994). V-ATPases containing these alternate a-subunits differ in proton pump energy-coupling efficiency, subcellular localization and in the regulation of subunit association in response to metabolic changes (Kawasaki-Nishi et al., 2001). In mammals, most V-ATPase subunits exist in multiple isoforms that are expressed in a tissue-specific manner, and isoform-specific activities or localization have been demonstrated in many species. For example, there are four a-subunit isoforms (a1–a4), which direct their cognate complexes to distinct cellular destinations (Chen et al., 2024; Chu et al., 2021; Matsumoto et al., 2018; Sun-Wada and Wada, 2013, 2022; Sun-Wada et al., 2007; Toei et al., 2010). An extreme example of such isoform-specific targeting comes from the ciliate *Paramecium tetraurelia*, in which 17 a-subunit isoforms localize to at least seven different compartments (Wassmer et al., 2006).

Notwithstanding the importance of compartment-specific optimization, the conserved activity of V-ATPases is proton pumping. However, several recent studies have identified non-acidic LROs in different cell types. For example, melanosomes in melanocytes are only transiently acidic: immature melanosomes are acidic, whereas mature melanosomes are non-acidic, which might be more consistent with their role as storage rather than degradative compartments (Correia et al., 2018; Hurbain et al., 2018; Le et al., 2021). Similarly, LAMP1-positive but non-acidic and non-degradative endolysosome-related organelles (ELROs) are found in neuronal cells (Cheng et al., 2018; Yap et al., 2018). In *Dictyostelium discoideum*, LROs called post-lysosomes originate as acidic lysosomes but subsequently lose their acidity and mature into neutral pH organelles (Fok et al., 1993; Padh et al., 1993). The mechanisms involved in the neutralization of these organelles in widespread eukaryotic lineages are not known.

One organism with non-acidic LROs is the ciliate *P. tetraurelia*, where secretory LROs called trichocysts dock at the plasma membrane to release their contents via exocytosis in response to extracellular stimuli (Plattner, 2017). However, V-ATPase activity nonetheless appears to be required for trichocyst formation (de Loubresse et al., 1994; Lumpert et al., 1992). To understand this paradox and shed light on the nature of neutral pH LROs, we have extended the analysis in *Paramecium* using the related oligohymenophorean ciliate *Tetrahymena thermophila*, where the homologous organelles to trichocysts are known as mucocysts (Turkewitz, 2004). Mucocysts, like LROs in many organisms, undergo biochemical and morphological maturation (Briguglio et al., 2013; Kaur et al., 2017; Kumar et al., 2014, 2015; Sparvoli et al., 2018). The most abundant mucocyst cargo proteins are encoded by two multigene families: GRL (granule lattice) and GRT/IGR (granule tip/induced on granule regeneration) (Bowman et al., 2005a,b; Cowan et al., 2005). Grl proteins are synthesized as proproteins and are subsequently cleaved and trimmed by processing enzymes including cathepsin 3 (Cth3p, where ‘p’ denotes the protein; encoded by *CTH3*) to generate mature Grls (Kumar et al., 2014, 2015).

Importantly, cargo delivery to mucocysts involves at least two different pathways. The delivery of Cth3p and Grt/Igr proteins, but not Grls, requires the sortilin/VPS10 receptor Sor4p and an endosomal SNARE, Stx7l1p (syntaxin 7-like) (Briguglio et al., 2013; Kaur et al., 2017; Sparvoli et al., 2018). The Grl and Grt/Igr proteins localize to different vesicles during mucocyst formation; these and other data argue that heterotypic fusion between secretory and endosomal vesicles is a key step in formation of ciliate LROs, like in other lineages. In *Tetrahymena* that fusion requires Vps8ap, a subunit of a dedicated class C core endosomal vacuole tethering (CORVET) complex (Sparvoli et al., 2018). The formation of mucocysts also requires the AP-3 complex, and cells lacking the AP-3µ subunit accumulate abortive mucocyst intermediates distinct from those in *VPS8A* knockout cells (Kaur et al., 2017).

In this study, we confirmed that mature mucocysts, like trichocycsts, are not acidified. Nonetheless, their biogenesis depends on the V-ATPase a1-subunit isoform, one of six a-subunit paralogs/isoforms in *T. thermophila*, which localizes to a variety of compartments including both biosynthetic mucocyst intermediates as well as mature docked mucocysts. By targeting a pH sensor to the mucocyst lumen, we found evidence that mucocysts are transiently acidified during their formation. Consistent with this, we found that three other subunits of the V-ATPase are also present at that stage in mucocyst formation. Analysis of cells lacking V-ATPase a1-subunit expression suggests that the failure to acidify during mucocyst maturation leads to defects in both proprotein (Grl) processing and the heterotypic vesicle fusion characteristic of LRO formation. In striking contrast, several V-ATPase subunits are absent from mature docked mucocysts. Our findings suggest that the V-ATPase complex disassembles during mucocyst maturation and can therefore account for the neutral pH of the mature organelles.

## RESULTS

### Expression profiling reveals coregulation of mucocyst-associated proteins and the V-ATPase a1-subunit of the V_0_ domain of V-ATPase complex in *T. thermophila*

**Fig. 1.**
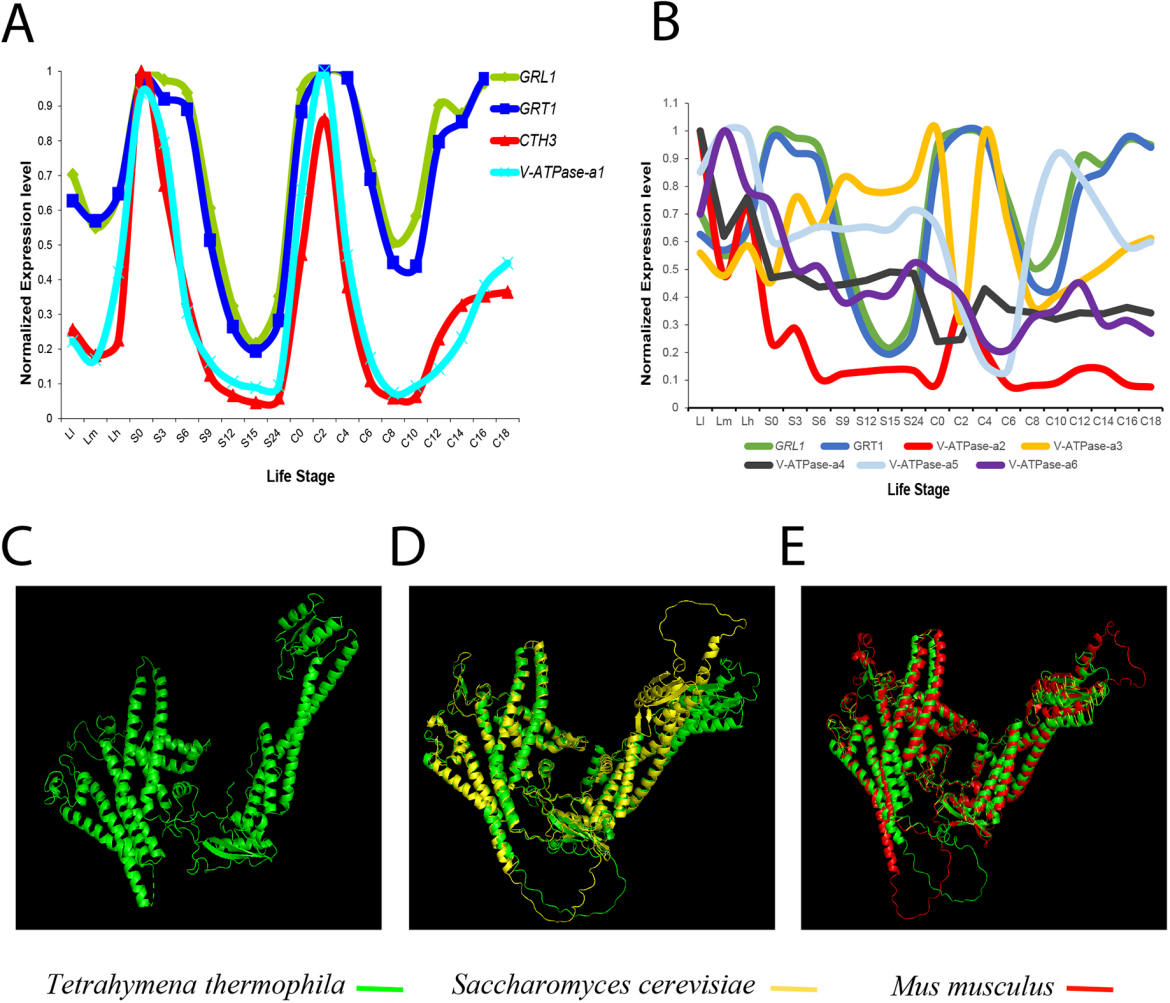
**The V-ATPase-a1 subunit of** ***T. thermophila*** **is coregulated with known mucocyst-associated genes and shares structural homology with V-ATPase-a1p subunits of other eukaryotes.** (A) Expression profiling identifies a subunit of the V*-*ATPase complex, V-ATPase-a1, that is transcriptionally coregulated with the known mucocyst-associated genes *GRL1*, *GRT1* and *CTH3*. The three major stages of the *Tetrahymena* life cycle are growth, starvation and conjugation. When food is abundant (growth condition), cells undergo into asexual reproduction through binary fission. The sexual stage, known as conjugation, is triggered by starvation. Transcript data for *V-ATPase-a1*, *GRL1*, *GRT1* and *CTH3* were retrieved from the Tetrahymena Functional Genomics Database, and the stages correspond to growth, starvation and conjugation (Ll, low density; Lm, medium density; Lh, high density; S, starvation; C, conjugation). For plotting the profiles, each value was normalized to the maximum expression level of the respective gene. (B) Other paralogs of the *V-ATPase-a* subunit (*a2*–*a6*) are not coregulated with known mucocyst genes. Data retrieved and plotted as in A. (C) The predicted structure of V-ATPase-a1p of *Tetrahymena* by the web portal Phyre2. (D,E) The predicted structure of V-ATPase-a1p of *T. thermophila* (green), *S. cerevisiae* (yellow) and *M. musculus* (red) by AlphaFold structure prediction. V-ATPase-a1p structure of *T. thermophila* superimposed with V-ATPase-a1p subunits of *S. cerevisiae* (D) and *M. musculus* (E) by PyMOL software.

The *T. thermophila* macronuclear genome has more than 26,000 predicted genes, including 11 that encode the subunits of the V_1_ domain and 12 that encode the subunits of the V_0_ domain of the V-ATPase complex (Coyne et al., 2008; Eisen et al., 2006; Ye et al., 2025). To ask whether there might be paralog-encoded subunits specialized for mucocysts, we followed up on earlier findings that many genes encoding mucocyst-related proteins are coregulated (Briguglio et al., 2013; Kumar et al., 2014; Miao et al., 2009; Xiong et al., 2011, 2013). We used the Tetrahymena Functional Genomics Database to identify V-ATPase subunits that are coregulated with known mucocyst-associated genes. This search revealed that TTHERM_01332070, encoding an a-subunit of V-ATPase V_0_, was coregulated with known mucocyst-associated genes (Fig. 1A). We have named this paralog *a1*. The genome encodes five additional paralogs, which we named *a2* (TTHERM_00463420), *a3* (TTHERM_00266570), *a4* (TTHERM_00372530), *a5* (TTHERM_01005210) and *a6* (TTHERM_01014710). These paralogs are significantly diverged, with a maximum of 42% amino acid sequence identity to a1 (Fig. S1), and they show transcriptional profiles distinct from that of a1 (Fig. 1B).

*Tetrahymena* V-ATPase-a1p shares ∼30% sequence identity with human a-subunit isoforms (a1p, ATP6V0A1; a2p, ATP6V0A2; a3p, ATP6V0A3, also known as TCIRG1; and a4p, ATP6V0A4). The predicted structure of the *T. thermophila* V-ATPase-a1p was similar to those of V-ATPase a-subunits in other lineages (shown for opisthokonts) (Fig. 1C). Structures similar to the *T. thermophila* V-ATPase-a1p in the AlphaFold Protein Structure Database included a-subunits from *Saccharomyces cerevisiae* (Uniprot ID A0A7I9G792) and *Mus musculus* (Uniprot ID Q9Z1G4), the former with an 84% per-residue model confidence score (predicted local distance different test, pLDDT; Fig. 1D,E).

### V-ATPase-a1p localizes to mucocysts

**Fig. 2.**
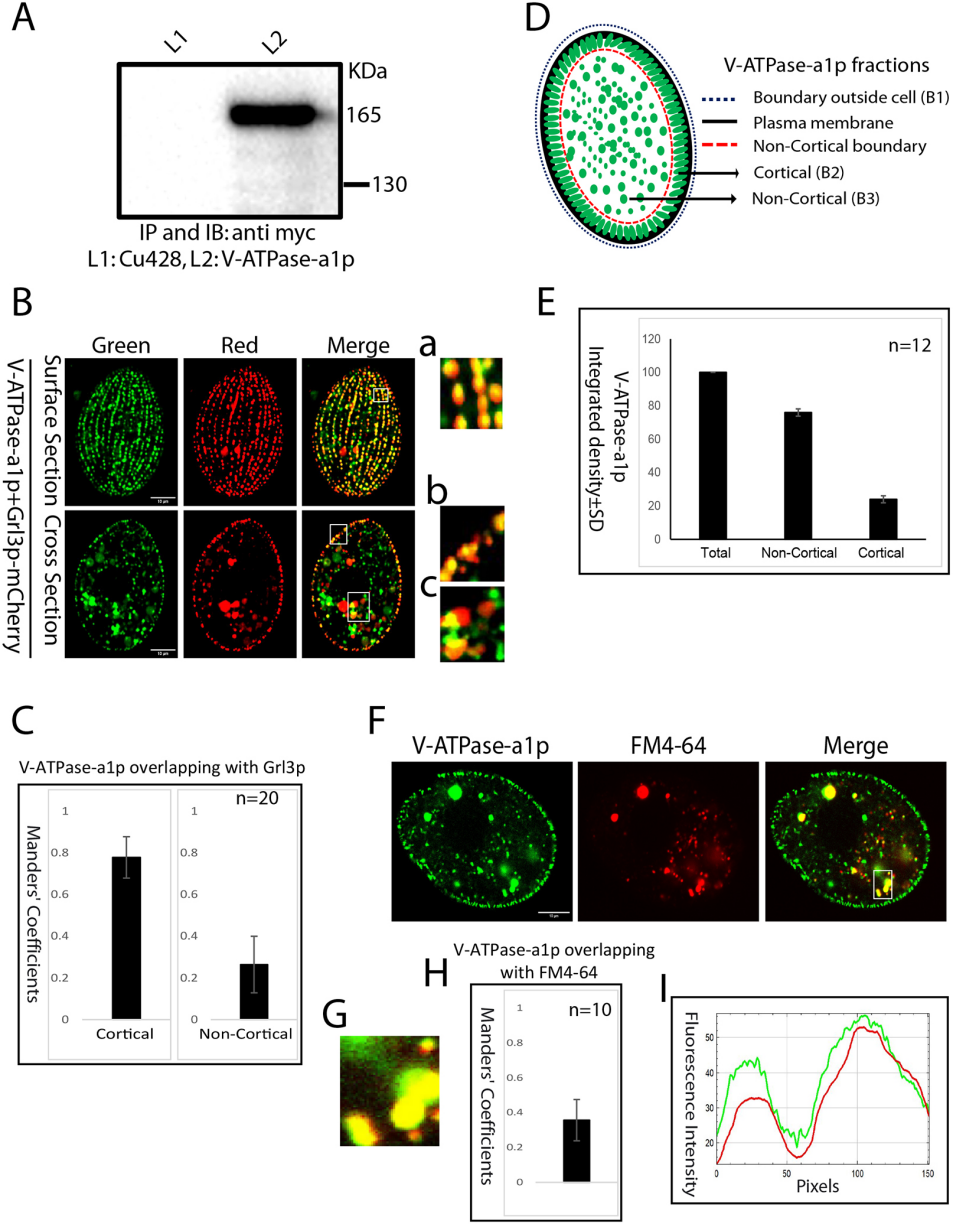
**Expression and localization of V-ATPase-a1p.** (A) Detergent solutes of wild-type cells (Cu428) and cells expressing V-ATPase-a1p–2×mNeon-6c-myc were immunoprecipitated (IP) using anti-c-Myc agarose beads. Pull-down samples were separated by SDS-PAGE and immunoblotted (IB) with mouse monoclonal anti-c-myc antibody. Blots were incubated with anti-mouse IgG–HRP for IP and developed using SuperSignal West Femto Maximum Sensitivity substrate. The expected size-specific band (∼164 kDa) was only found in the V-ATPase-a1p lane. Cells expressing V-ATPase-a1p–2×mNeon fusion protein are also tagged with c-Myc. Blot shown is representative of two independent experiments. (B) Cells co-expressing V-ATPase-a1p–2×mNeon (green) with the mucocyst marker Grl3p–3×mCherry (red), both at endogenous loci. V-ATPase-a1p and Grl3p overlap extensively at docked mucocysts. Images are shown as single slices of either surface sections or cross sections, for clarity. Images a, b and c are expanded views of the regions marked by boxes and depict colocalization of V-ATPase-a1p with Grl3p. To measure colocalization, the images were processed to remove noise and adjust the thresholds, with colocalization quantified using the Fiji-BIOP JACoP plugin. Scale bars: 10 μm. (C) A total of 20 cells as in B were used to calculate the overlap (expressed as the Manders’ coefficient) between V-ATPase-a1p and Grl3p in cortical (surface section) and non-cortical (cross section) domains. The error bars show the s.d. (D) Cartoon representing localization of V-ATPase-a1p at cortical and non-cortical compartments of cells. (E) The mean fluorescence intensity associated with cell (total, B1), cortical (B2) and non- cortical (B3) regions was measured and converted to integrated density. Most V-ATPase-a1p is found in non-cortical compartments. Mean±s.d. of *n*=12. Data are representative of three independent experiments. (F) V-ATPase-a1p in non-cortical puncta shows some overlap with endosomal marker FM4-64. Cells expressing V-ATPase-a1p–2×mNeon were incubated for 5 min with 5μM FM4-64 and then washed with 10 mM Tris-HCl (pH 7.4). Images were acquired after 30–40 min. Box marks the region shown as an expanded view in F. Scale bar: 10 μm. (G) Image showing the boxed region from F. (H) A total of 10 cells as in F were used to calculate the overlap (expressed as the Manders’ coefficient) between V-ATPase-a1p–2×mNeon and FM4-64 in non-cortical puncta. The error bars show the s.d. (I) Graph showing V-ATPase-a1p (green) and FM4-64 (red) fluorescence intensity along the length of the rectangle shown in F.

To ask whether the *Tetrahymena* a1-subunit localizes to mucocysts, we tagged the protein via C-terminal fusion of mNeonGreen (2×mNeon) at the endogenous locus. The expression in *Tetrahymena* of a fusion protein of the expected size was confirmed by immunoprecipitation followed by western blotting (Fig. 2A). Localization of V-ATPase-a1p to mucocysts was confirmed by expressing tagged V-ATPase-a1p in cells co-expressing mCherry-tagged mucocyst cargo protein Grl3p. The two proteins colocalized in the mucocysts at the cell cortex (Manders' coefficient 0.78±0.10, mean±s.d.), which based on their docking are assumed to be mature (Fig. 2B top and inset a, Fig. 2C left; Fig. S2A,B). In optical cross sections of the same cells, V-ATPase-a1p was also observed in heterogeneous cytoplasmic puncta removed from the cortex (which we call ‘non-cortical’). Approximately 75% of V-ATPase-a1p was present in the non-cortical compartments, with the remaining quarter located in the cortical docked mucocysts (Fig. 2B,D,E). The non-cortical factions of V-ATPase-a1p showed some overlap with Grl3p (Manders' coefficient 0.26±0.14, mean±s.d.) (Fig. 2B bottom and insets b, c, and Fig. 2C right; Fig. S2C). Since the Grl proteins appear exclusively localized to mucocysts, we interpret non-cortical structures containing Grl3p as likely vesicular intermediates in mucocyst biogenesis.

Strikingly, a large fraction of non-cortical V-ATPase-a1p was found in puncta that did not contain Grl3p. The non-cortical V-ATPase-a1p overlapped with the endocytic tracer FM4-64 (Manders' coefficient 0.36±0.12, mean±s.d.) (Fig. 2F–I), and to a lesser degree with the late endosomal marker Rab7p (Manders' coefficient 0.17±0.04, mean±s.d.) (Fig. S2D–G) or with Lysotracker (Manders' coefficient 0.08±0.04, mean±s.d.) (Fig. S2H–K). Collectively, the findings support the idea that non-cortical V-ATPase-a1p is associated with multiple structures including biosynthetic mucocyst intermediates, endosomes, and as-yet-undefined compartments. Based on their distinct expression profiles, the other V-ATPase-a1 paralogs might be specialized for other compartments. Consistent with this, we found that V-ATPase-a2p chiefly localized near the plasma membrane but not at Grl3p-positive mucocysts (Fig. S2L).

### Mucocysts are not stably acidic in *Tetrahymena*

**Fig. 3.**
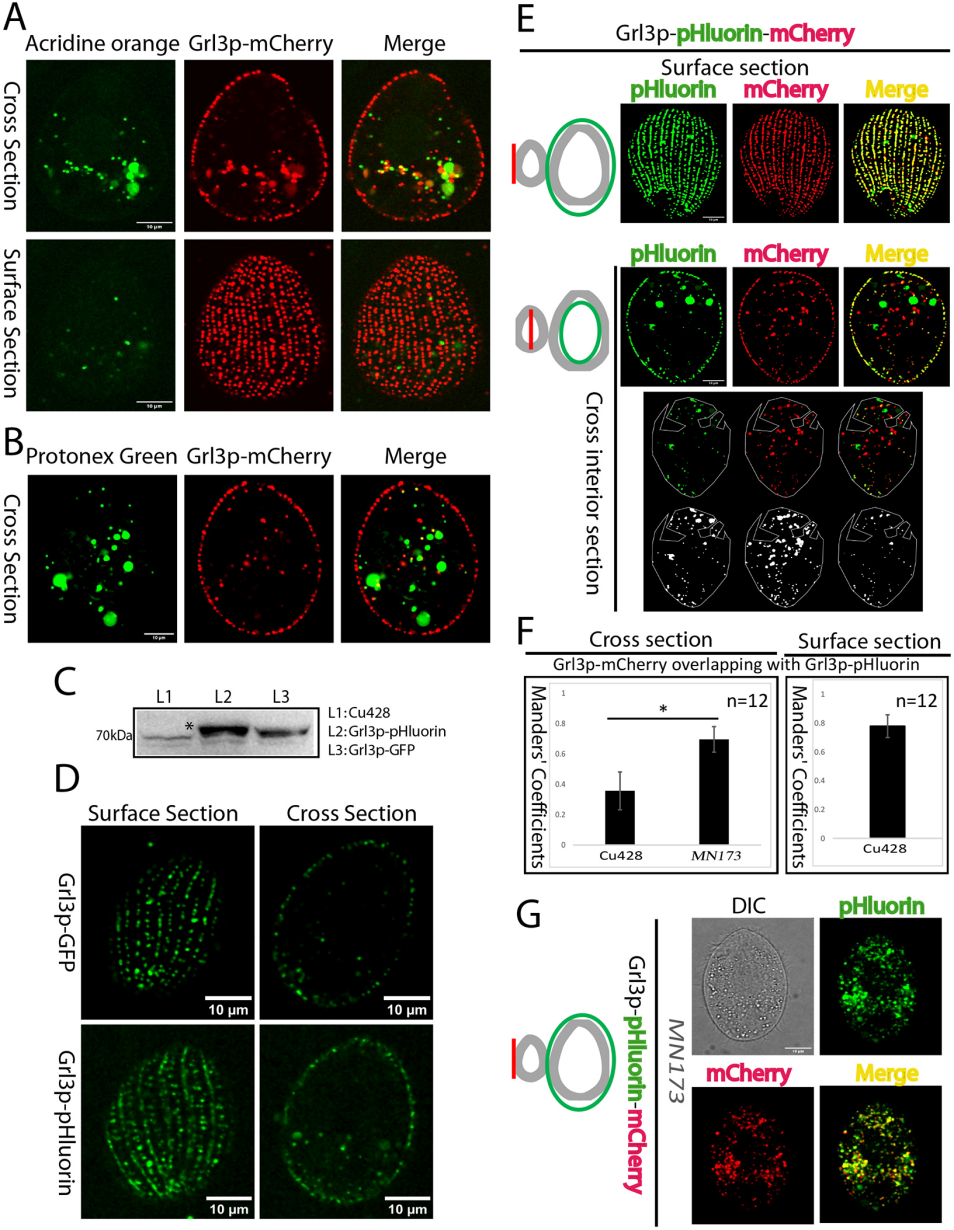
**The mucocyst lumen is not stably acidic.** (A) Cells expressing mucocyst cargo Grl3p–3×mCherry incubated with Acridine Orange. There is minimal overlap between Acridine Orange and Grl3p in docked mucocysts. Confocal images are shown as single slices, corresponding to cross sections or surface sections as indicated, for clarity. Images are representative of three independent experiments. (B) Cells expressing mucocyst cargo Grl3p–3×mCherry were incubated with Protonex Green. No substantial colocalization is seen between Protonex Green and Grl3p in docked mucocysts. Images are representative of three independent experiments. (C) Cell lysates from the indicated cell lines (60,000 cell equivalents; Cu428, wild-type cells) were separated using SDS-PAGE, transferred to PVDF and probed with anti-GFP mAb, which also recognizes pHluorin. Bands corresponding to Grl3p–GFP and Grl3p–pHluorin are indicated by an asterisk. Blot shown is representative of two independent experiments. (D) Grl3p–GFP and Grl3p–pHluorin constructs were expressed at the native *GRL3* locus. The super-ecliptic pHluorin (SEP) emits fluorescence at pH 6.0 or higher. Both Grl3p–GFP and Grl3p–pHluorin show strong signals in docked mucocysts, consistent with their characterization as a non-acidic compartment. Images are representative of three independent experiments. (E–G) Endogenous expression of Grl3p–pHluorin–mCherry in Cu428 (wild-type) and mutant *MN173* cells. (E) In wild-type cells, dual emission from both pHluorin and mCherry at each fluorescent spot was measured as colocalization, and was analyzed in cell surface sections (top) and cross sections (middle). The images were processed to remove noise and adjust thresholds, with colocalization quantified using the Fiji BIOP JACoP plugin. The edges of the cross sections were excluded (marked by a green boundary line in the illustration on the left) to restrict analysis to non-cortical structures. To minimize false colocalization due to the bright autofluorescence from large food vacuoles, we selected areas lacking food vacuoles (bottom) for quantitative analysis. The color images at the bottom illustrate colocalization in selected non-cortical regions (marked by the white boundary line), while the gray images reveal colocalization after threshold adjustments for the green and red channels. Surface sections were analyzed to measure colocalization in cortical structures. The pHluorin and mCherry signals showed complete overlap within the docked mucocysts. There was also some colocalization of red and green signals in non-cortical structures, as shown in cross sections of the same cells. (F) Twelve cells (three independent experiments) as in E and G were analyzed for non-cortical (left) and cortical (right) puncta. A two-tailed unpaired Student's *t*-test was performed between wild-type and *MN173* cells for colocalization of pHluorin and mCherry (expressed as the Manders’ coefficient) in non-cortical compartments using MedCalc statistical software. *P<0.0001. The error bars show the s.d. (G) Colocalization was measured as shown in E for cross sections of *MN173* cells. Significant overlap of green and red signals was observed in the non-cortical region (see F). DIC, differential interference contrast. Scale bars: 10 μm.

*Paramecium* trichocysts, which are homologous to *Tetrahymena* mucocysts, are not detectibly acidic (de Loubresse et al., 1994; Lumpert et al., 1992). However, the finding that a key protease required for *Tetrahymena* mucocyst formation is related to lysosomal enzymes suggests that mucocyst biogenesis requires acidification (Kumar et al., 2014). Therefore, we asked whether the mucocyst lumen is acidic. First, we incubated cells expressing Grl3p–mCherry with Acridine Orange dye, which accumulates in acidic vesicles. There was no visible accumulation of Acridine Orange at docked mucocysts (Fig. 3A). Similarly, we incubated cells expressing Grl3p–mCherry with an alternative probe for acidic compartments, Protonex Green (Prakash et al., 2021). Consistent with the Acridine Orange results, we observed no concentration of Protonex Green in mucocysts at the cell cortex (Fig. 3B).

We took a third and more flexible approach to analyzing the pH of mucocysts by endogenously tagging Grl3p with either mEGFP or the super-ecliptic pHluorin (SEP) variant, a green fluorescent protein whose emission is relatively quenched at acidic pH. In mammalian cells in which SEP is targeted to the lumen of acidified secretory vesicles, SEP emits bright fluorescence only after the vesicles undergo exocytosis, when their contents become neutralized (Benčina, 2013; Martinière et al., 2013; Michaluk and Rusakov, 2022). In our experiments, the salient advantage to using tagged Grl3p as a pH sensor is that it also allowed us to analyze the pH in the lumen of non-docked, biosynthetic mucocyst intermediates.

We first confirmed the expression of the fusion proteins Grl3p–mEGFP and Grl3p–pHluorin by western blotting using an anti-GFP monoclonal antibody (mAb) (Fig. 3C). Importantly, by confocal microscopy we detected strong fluorescence signals for both Grl3p–mEGFP and Grl3p–pHluorin in docked mucocysts (Fig. 3D). Since the pHluorin signal in the easily identified docked mucocysts was not quenched, this result provides additional confirmation that mature mucocysts are not acidified. Since we could also detect non-quenched cytoplasmic pHluorin signal in non-cortical puncta, some mucocyst intermediates might also be non-acidic.

To address the pH of mucocyst intermediates more rigorously, we endogenously tagged Grl3p in tandem with pHluorin followed by mCherry, whose fluorescence emission is not pH sensitive (Doherty et al., 2010; Hundeshagen et al., 2011; Shaner et al., 2004, 2005). By simultaneously collecting data in the red and green channels, we could detect acidic mucocysts (mature or intermediates) as puncta showing red but not green fluorescence. In the docked mucocysts of wild-type cells (i.e. the cortical field), the large majority of red puncta also showed green fluorescence, with the overlap between signals having a Manders' coefficient of 0.78±0.088 (mean±s.d.; Fig. 3E top and Fig. 3F right). In contrast, the overlap between green and red signals was lower in non-cortical puncta (Manders' coefficient 0.36±0.13, mean±s.d.; Fig. 3E middle and bottom, and Fig. 3F, left). Thus, there is a significant population of Grl3p-containing puncta in the cytoplasm in which the pHluorin emission is quenched. This result is consistent with the idea that a sub-population of mucocyst intermediates are acidic, which can be most simply explained if mucocysts are transiently acidified during their formation.

**Fig. 4.**
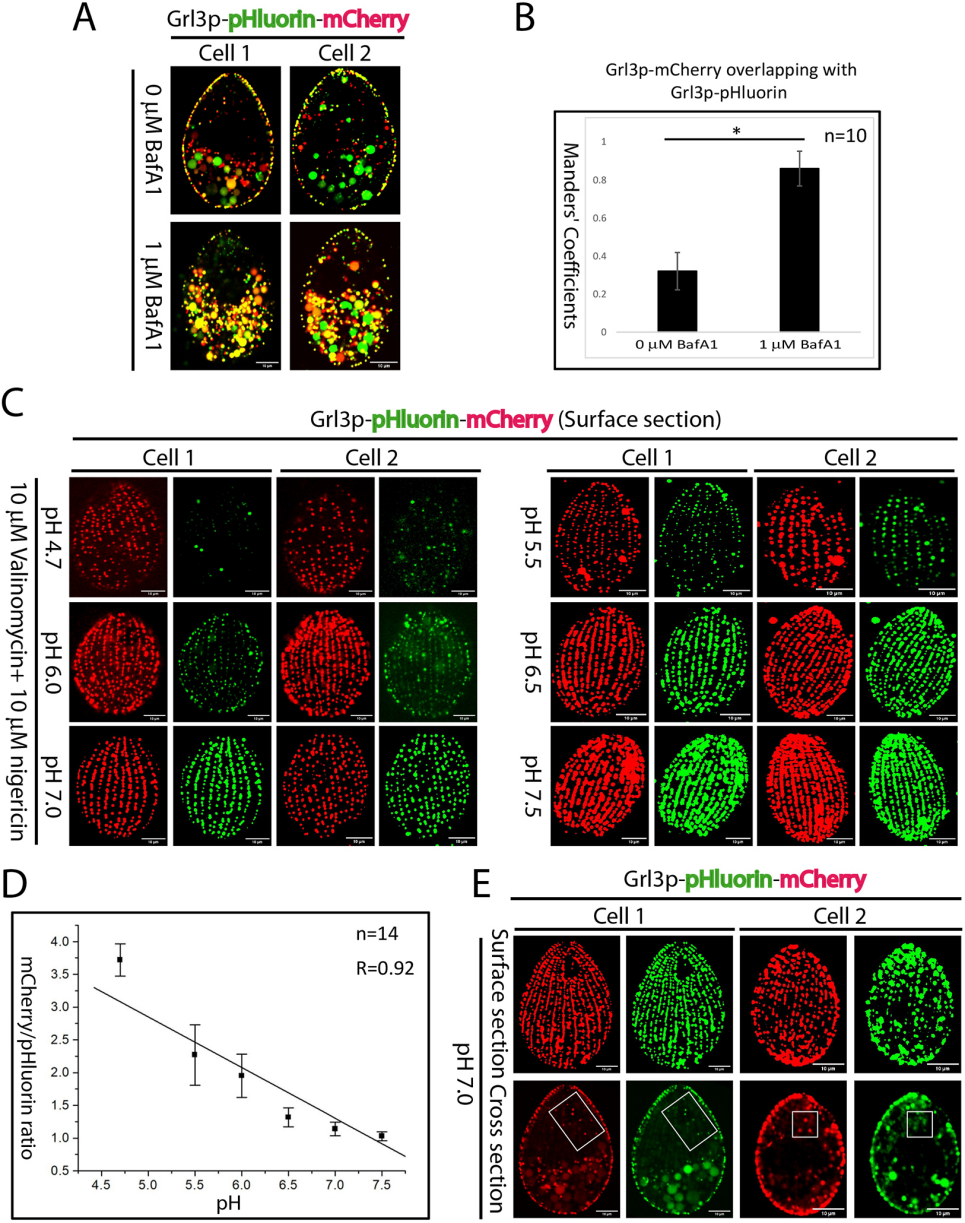
**Mucocysts are transiently acidified during their formation in** ***T. thermophila*****.** (A,B) Cells that expressed Grl3p–pHluorin–mCherry showed a nearly complete overlay of pHluorin and mCherry signals after BafA1 treatment. (A) Cells were grown to reach mid density and subsequently treated with 1% DMSO or 1% DMSO containing 1 µM BafA1 for 16 h before images were captured. (B) Colocalization was measured in cross interior sections from 10 cells as in A using the Fiji BIOP JACoP plugin. Colocalization is expressed as the Manders’ coefficient, and the error bars show the s.d. *P<0.0001 (two-tailed unpaired Student's *t*-test). (C) Grl3p–pHluorin–mCherry-expressing cells were starved for 2 h in DMC buffer (pH 7.0) with 100 mM KCl and treated with ionophores (nigericin and valinomycin) in different pH buffers for 5 min, and then images were captured as before. (D) For each pH condition, 14 cells as in C were used to calculate the mean fluorescence intensity for both pHluorin and mCherry using Fiji. The graph shows the mean±s.d. ratio of mCherry MFI to pHluorin MFI at various pH levels post-ionophore treatment. R, correlation coefficient. (E) Grl3p–pHluorin–mCherry-expressing cells were placed in DMC buffer for starvation, images were captured and the ratio of mCherry MFI to pHluorin MFI was measured from 17 cells. Surface sections and cross sections were used to measure the pH of cortical and non-cortical compartments of mucocysts, respectively. In the cross section, the marked rectangular regions were used to measure the pH of non-cortical Grl3p-positive compartments. Scale bars: 10 μm. Images are representative of three independent experiments.

To test our hypothesis that the pHluorin fluorescence linked with Grl3p was quenched in a subset of mucocyst intermediates, we treated cells expressing Grl3p–pHluorin–mCherry with bafilomycin A1 (BafA1), a specific and potent inhibitor of V-ATPases. BafA1 disrupts the proton gradient required for maintaining acidic environments within organelles (Wang et al., 2021). In untreated cells, mCherry-positive non-cortical puncta showed some overlap with pHluorin signals (Manders' coefficient 0.32±0.09, mean±s.d.; Fig. 4A,B). Strikingly, following BafA1 treatment there was nearly complete overlap between the green and red signals (Manders' coefficient 0.86±0.09, mean±s.d.; Fig. 4A,B). These data are strongly consistent with the model that a cohort of cytoplasmic mucocysts are acidified at a transient stage in their formation. Our data could also be explained if mucocyst docking per se caused neutralization of the luminal pH. To examine this possibility, we exploited a *Tetrahymena* mutant, strain *MN173*, in which mucocysts undergo normal biochemical and morphological maturation but remain dispersed in the cytoplasm rather than docking (Chilcoat et al., 1996; Melia et al., 1998). Although the mutation responsible for this phenotype has not yet been mapped, *MN173* provided us with a case in which luminal neutralization and cortical localization should be decoupled, differently to situation in wild-type cells. Indeed, *MN173* cells expressing Grl3p–pHluorin–mCherry showed complete colocalization of pHluorin and mCherry in cytoplasmic puncta (Fig. 3F left and Fig. 3G). These results are consistent with the idea that the limited overlap of green and red signals in the cytoplasmic puncta of wild-type cells is not an artifact and instead reflects a population of acidified mucocyst intermediates.

To directly measure the pH of mucocysts, we treated cells with ionophores (nigericin and valinomycin) in various pH buffers and measured the ratio of mCherry mean fluorescence intensity (MFI) to pHluorin MFI at the cell cortex, where mature mucocysts are docked (Fig. 4C,D). Our results showed that the pH of cortical puncta containing Grl3p–pHluorin–mCherry was ∼7.2±0.06 (mean±s.d.; Fig. 4E top). To measure the pH of mucocyst intermediates in the non-cortical cytoplasm, we focused within cell cross sections on Grl3p-positive puncta within regions that appeared to be free of degradative compartments (food vacuoles or multivesicular structures). We found that the pH of these puncta was acidic (∼5.6±0.28, mean±s.d.; Fig. 4E bottom).

### The holo-V-ATPase complex does not persist during mucocyst maturation

The data shown above suggest that V-ATPase-a1p is present in transiently acidified mucocyst precursors but is also present in docked mucocysts, which no longer maintain a pH gradient. We therefore asked whether other subunits of the V-ATPase complex were similarly localized, or instead whether V-ATPase composition might change during mucocyst maturation. For this work, it was important to avoid ambiguities from studying genes that have multiple paralogs. Approaches including BLAST searches (https://blast.ncbi.nlm.nih.gov/Blast.cgi) and structural homology revealed that V_1_ subunits including Vma1p (subunit A), Vma8p (subunit D) and Vma10p (subunit G) are present as single genes in *Tetrahymena* (Table S1, Fig. S3A–E). The *T. thermophila* Vma1p, Vma8p and Vma10p homologs have high sequence identity with Vma subunits in *S. cerevisiae* (Table S1, Fig. S3A–E). Consistent with the sequence identity, the Phyre2-predicted structures of Vma1p and Vma8p revealed high structural similarity with subunit A and subunit D of the V_1_ domain of mammalian cells, respectively (Fig. S3A,B). *T. thermophila* Vma10p also showed predicted structural similarity to a V-ATPase subunit in *S. cerevisiae*, subunit G, though this was more limited (62% coverage and 28% structural identity; Fig. S3C). The predicted structural similarities between *T. thermophila* and human V-ATPase subunits were also supported by AlphaFold predictions for subunit A (Fig. S3D) and subunit D (Fig. S3E) of *T. thermophila* (green) and human (blue) V-ATPase. Taken together, these sequence and structural similarities strongly argue that these genes in *T. thermophila* encode components of the proton-translocating V-ATPase complex.

**Fig. 5.**
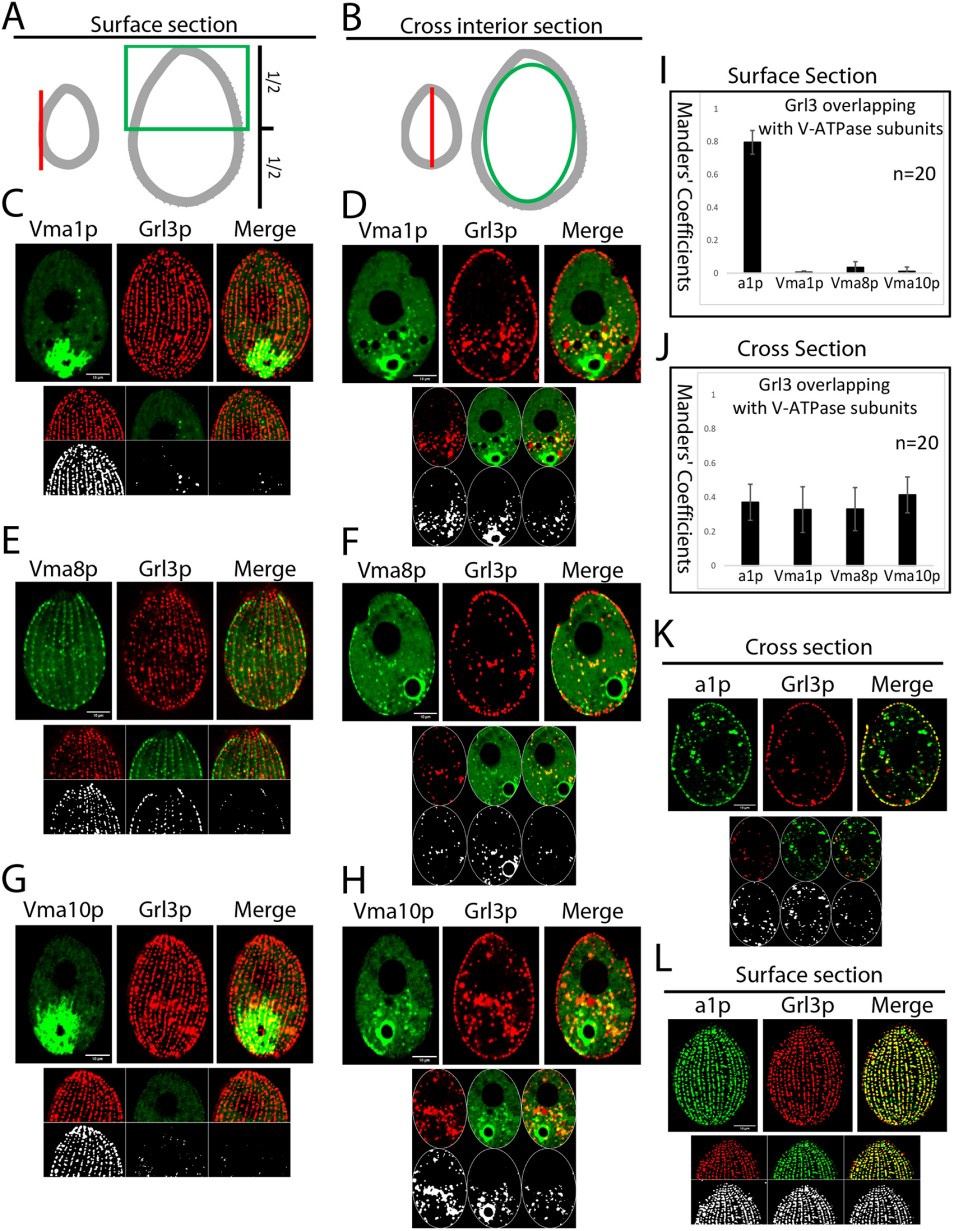
**The V-ATPase complex in mature, but not immature, mucocysts lacks multiple subunits.** (A,B) The colocalization of Vma subunits and Grl3p was measured using cell surface sections (A) and cross sections (B). For the cross sections, the edge of the sections was excluded (indicated by green boundary line in cartoon, panel B) in order to measure colocalization in non-cortical structures. The surface sections (panel A) were used to measure colocalization in cortical structures. To reduce false colocalization in cortical sections due to the bright signal from the contractile vacuole near the cell posterior, only the region approximately corresponding to the anterior half of the cell was analyzed. (C–H) Cells co-expressing 2×mNeon-tagged Vma1p (C,D), Vma8p (E,F) or Vma10p (G,H) with the mucocyst marker Grl3p–3×mCherry, all at their endogenous loci. In both cross sections and surface sections, Vma subunits are localized at the contractile vacuoles and in non-cortical puncta, as well as at the cell cortex. Colocalization was measured using the Fiji BIOP JACoP plugin as shown in A and B. The color images at the bottom of each panel show colocalization in selected non-cortical (cross section) and cortical (surface section) compartments. Gray images show colocalization after adjusting the thresholds for the green and red channels. There was no colocalization of Vma subunits with Grl3p in mature, docked mucocysts at the cell cortex. In contrast, there was less overlap between Grl3p and the Vma subunits in non-cortical compartments. (I,J). For each of C–H, K and L, 20 cells were used to calculate the overlap (expressed as the Manders’ coefficient) between Grl3p and V-ATPase subunits (Vma1p, Vma8p, Vma10p and V-ATPase-a1p) in cortical (I) and non-cortical compartments (J). The error bars show the s.d. (K,L) Co-expression of V-ATPase-a1p–2×mNeon and Grl3p–3×mCherry. In the surface section (L), there was complete overlap between Grl3p and V-ATPase-a1p in the docked mucocysts. There was also some colocalization of V-ATPase-a1p and Grl3p in non-cortical structures, as seen in cross sections (K) of the same cells. Images at the bottom of each panel show colocalization, as described for C–H. Scale bars: 10 μm.

For each of these three V-ATPase subunits in *Tetrahymena*, we asked whether they were present in mucocysts. We imaged cells that endogenously co-expressed Grl3p–mCherry with mNeonGreen-tagged Vma1p, Vma8p or Vma10p (Fig. 5A–J), and measured colocalization using the Fiji BIOP JACoP plugin and fluorescence intensity line profile plot in surface sections (Fig. 5A) and cross sections (Fig. 5B). We found negligible overlap between the Vma subunits and Grl3p in cortical mucocysts (Fig. 5C,E,G,I; Fig. S3F,H,J), in contrast to the expected extensive overlap between Grl3p and V-ATPase-a1p (Fig. 5I,K; Fig. S3L).

Strikingly, the distribution of Vma subunits at non-cortical Grl3p-positive puncta was very different from that at the cortex, with obvious overlap between Grl3p and all V-ATPase subunits (Vma1p, Vma8p, Vma10p and, as expected, V-ATPase-a1p) (Fig. 5D,F,H,J,L; Fig. S3G,I,K,M). These results argue that three subunits of the V-ATPase complex are present in mucocyst intermediates but not mature mucocysts. We cannot rule out the possibility that some Grl3p-positive puncta could represent degradative, rather than biosynthetic, compartments, but previous studies of Grl proteins provide no evidence for their targeting to late endosomes or lysosomes. Those three Vma subunits, since they have no paralogs, would also be expected to participate in a variety of V-ATPase complexes that do not contain V-ATPase-a1p. We endogenously co-expressed 2×mNeon-tagged V-ATPase-a1p with Vma1p–3×mCherry and found the expected partial overlap in non-cortical puncta (Manders' coefficient 0.24±0.06, mean±s.d.; Fig. S4A–E). This was similar to the overlap in non-cortical puncta between V-ATPase-a1p and Grl3p (Fig. 2B and Fig. 2C right).

Taken together, these results argue that at least four subunits of the enzymatically active holo-V-ATPase complex are present in a population of cytoplasmic mucocysts that are undergoing maturation. However, only a subset of those subunits are found in mature mucocysts docked at the cell surface. These results suggest that disassembly of the V-ATPase complex occurs during mucocyst maturation. The incompleteness of the V-ATPase complex present in mature mucocysts provides an explanation for why these organelles do not maintain an acidic lumenal environment. Our result is consistent with a recent study that showed subunit G of the mammalian V_1_ domain, homologous to the Vma10p subunit, disassembles reversibly from lysosomes under certain conditions (Sava et al., 2024).

### *V-ATPase-a1* is essential for mucocyst formation

**Fig. 6.**
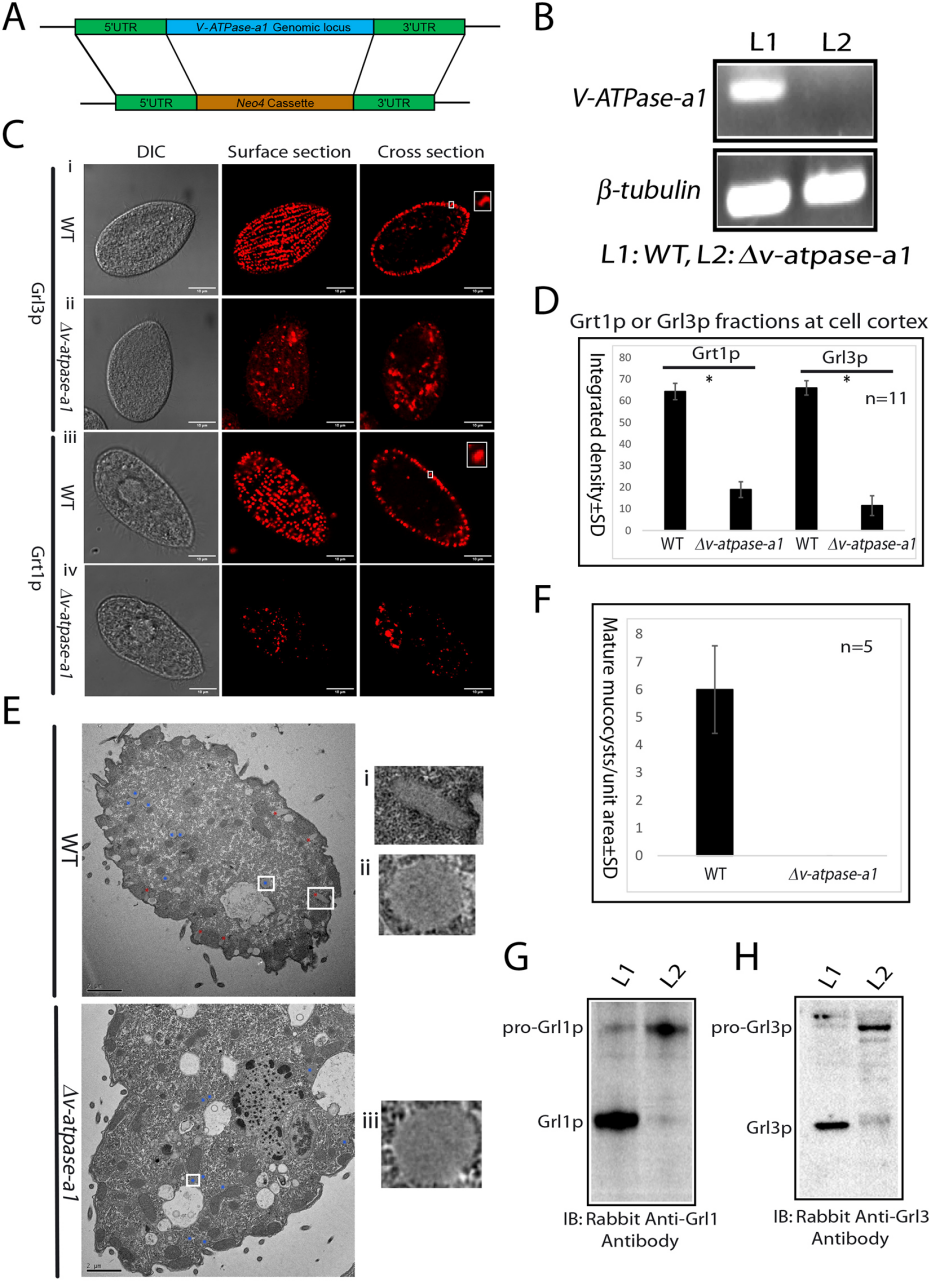
**Analysis of the role of *V-ATPase-a1* in mucocyst biogenesis.** (A) *V-ATPase-a1* knockout by homologous recombination. The knockout construct is described in the Materials and Methods. (B) Confirmation of *V-ATPase-a1* knockout by one-step RT-PCR. PCR primers are listed in Table S4. *BTU2* (*β-tubulin*) was used as a control. WT, wild type. Data shown are representative of three independent experiments. (C–F) V-ATPase-a1p is essential for mucocyst formation. (C) Docked mucocysts visualized in fixed wild-type cells by anti-Grl3p mAb 5E9 (panel i) and anti-Grt1p mAb 4D11 (panel iii). *Δv-atpase-a1* cells contain less mucocyst signal, and the Grl3p puncta are often irregularly shaped (panels ii and iv). Insets show the single docked mucocysts marked by boxes in the main images. The 5E9 antibody recognized both immature and mature mucocysts. Scale bars: 10 μm. DIC, differential interference contrast. (D) A total of 11 cells as in C were used to measure mean fluorescence intensity of Gr13p and Grt1p at the cell cortex, which was converted to integrated density as described for Fig. 2E. The error bars show the s.d. *P<0.0001 (two-tailed unpaired Student's *t*-test). (E) Electron micrographs show mucocysts in a wild-type (top) and *Δv-atpase-a1* (bottom) cell. In the wild-type cell, immature mucocysts (labeled with blue asterisks) and docked mature mucocysts (labeled with red asterisks) are present. Immature mucocysts are morphologically heterogenous but less elongated and located deeper within the cytoplasm, compared to mature mucocysts, which are chiefly docked at the plasma membrane. In the *Δv-atpase-a1* cell, no mature mucocysts are present. Boxes indicate regions shown as magnified views (i, ii and iii). Scale bars: 2 μm. Images are representative of five cells. (F) Electron micrographs from five cells as in E were used to quantify mature mucocysts at the cell cortex in each unit area. The error bars show the s.d. (G,H) V-ATPase-a1p is required for processing of Grl proproteins. Cell lysates (5000 cell equivalents in G and 10,000 cell equivalents in H) were separated by SDS-PAGE and western blotted using antibodies against Grl1p and Grl3p. (G) Western blotting using rabbit anti-Grl1p antibody. The mature processed Gr1p product predominates in wild-type cells, while the unprocessed precursor of Grl1p predominates in *Δv-atpase-a1* cells. (H) Same as G but using anti-Grl3p antibody. Lane L1, wild type; lane L2, *Δv-atpase-a1*. Blots shown are representative of two experiments.

To ask whether *V-ATPase-a1* is required for mucocyst biogenesis, we subcloned the 5′ untranslated region (UTR) and 3′ UTR of the *V-ATPase-a1* gene into a pNeo4 knockout vector and transformed it into *Tetrahymena* by biolistic transformation to target the *V-ATPase-a1* gene for disruption via homologous recombination with a drug-resistance cassette (Fig. 6A). During ∼3–4 weeks of selection, all ∼45 copies of a gene in the polyploid macronucleus can be replaced with the cassette, resulting in a functional knockout if the gene is non-essential (Coyne et al., 2008). After generating the *V-ATPase-a1* knockout line, called Δ*v-atpase-a1*, we used reverse transcription PCR (RT-PCR) to detect the gene transcript. The *V-ATPase-a1* transcript was not found in the deletion line (Fig. 6B), which grew at wild-type rates under standard laboratory conditions. Hence the gene can be considered non-essential.

To test whether *V-ATPase-a1* was required for mucocyst formation, we used indirect immunofluorescence with two mAbs that recognize, respectively, Grl3p (mAb 5E9) and Grt1p (mAb 4D11) (Cowan et al., 2005; Kumar et al., 2014). Imaging of wild-type cells using either antibody brightly illuminates the mucocysts primarily docked at the cell periphery, as seen in cell cross sections (Fig. 6Ci, iii). In contrast, in *Δv-atpase-a1* cells both Grl3p and Grt1p were relatively sparse, and no mucocysts were docked at the cortex (Fig. 6Cii, iv). Moreover, a lower signal intensity was observed in *Δv-atpase-a1* cells compared to wild-type cells. Notably, large fractions of the Grl3p and Grt1p in the *Δv-atpase-a1* cells are distant from the cell cortex (Fig. 6Cii, iv and Fig. 6D). *V-ATPase-a1* therefore is required for mucocyst formation. We also examined wild-type and *Δv-atpase-a1* cells using electron microscopy (Fig. 6E; Fig. S5). In wild-type cells, both immature mucocysts, as well as mature mucocysts docked at the cell cortex, were detected (Fig. 6Ei, ii and Fig. 6F). In the *Δv-atpase-a1* mutant, only structures resembling immature mucocysts were observed (Fig. 6Eiii and Fig. 6F; Fig. S5).

### *V-ATPase-a1* is required for the processing of pro-Grl proteins

The dense lumenal proteinaceous core of mucocysts in wild-type cells is composed of Grl proteins, and the formation of the mucocyst core depends upon their proteolytic processing (Cowan et al., 2005; Turkewitz et al., 1991). The morphologically aberrant mucocysts in *Δv-atpase-a1* cells might be caused by defective processing of Grl proteins (Fig. 6C–F). Therefore, we asked whether *V-ATPase-a1* is required for Grl proprotein processing. We analyzed whole-cell lysates of the *Δv-atpase-a1* cells by western blotting using anti-Grl antisera (Fig. 6G,H). In the mutant cells, the Grl proteins were found to accumulate as unprocessed or partially processed precursors (Fig. 6G,H).

### *V-ATPase-a1* is required for a key membrane trafficking step during mucocyst formation

In addition to proteolytic maturation of Grl proproteins, a well-documented step in mucocyst formation is heterotypic vesicle fusion that brings together the Grl proteins, which might be trafficked directly from the Golgi or *trans*-Golgi network (TGN), with Grt/Igr proteins as well as at least some key Grl proprotein processing enzymes, which might be trafficked via endosomes. This fusion step involves the syntaxin 7-like homolog Stx7l1p and the CORVET subunit Vps8ap (Sparvoli et al., 2018). Stx7l1p itself, but not Vps8ap, remains associated with mature mucocysts. In cells lacking either *VPS8A* or *STX7l1*, Grl and Grt/Igr proteins accumulate in separate vesicles.

**Fig. 7.**
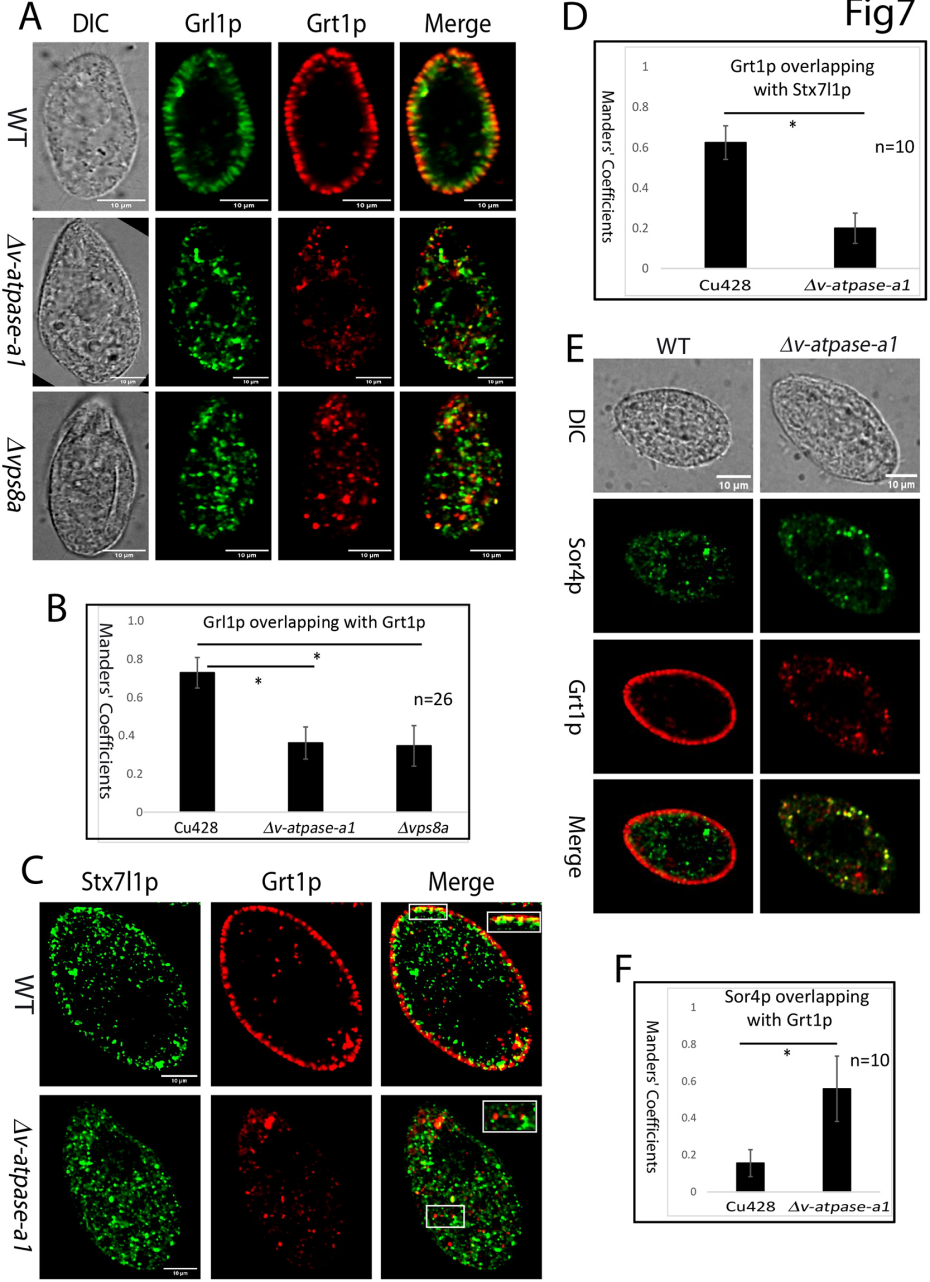
**The *Δv-atpase-a1* cells show defects in vesicle trafficking during mucocyst biogenesis, similar to those in *Δvps8a* cells.** (A) Wild-type (WT, Cu428; top), *Δv-atpase-a1* (middle) and *Δvps8a* (bottom) cells were double stained with rabbit anti-Grl1 and mouse monoclonal anti-Grt1 (4D11) antibodies. Colocalization of Grl1p and Grt1p is significantly reduced in *Δv-atpase-a1* and *Δvps8a* cells, compared to that in wild-type cells. Optical cross sections are shown as single slices, for clarity. (B) Overlap (expressed as the Manders’ coefficient) between Grl1p and Grt1p using a sample of 26 cells as in A. The error bars show the s.d. (C) Wild-type (top) and *Δv-atpase-a1* (bottom) cells expressing Stx7l1p–GFP were double stained with rabbit anti-GFP and mouse monoclonal anti-Grt1(4D11) antibodies. In *Δv-atpase-a1* cells, there is a significant decrease in the colocalization of Stx7l1p and Grt1p. Inset shows colocalization between Stx7l1p and Grt1p for the regions marked by boxes. (D) A field-of-view from ten cells as in C was analyzed to determine the overlap (expressed as the Manders’ coefficient) between Stx7l1p and Grt1p using the Fiji-JACoP plugin. The error bars show the s.d. (E) Wild-type (left) and *Δv-atpase-a1* (right) cells expressing Sor4p–GFP were double stained with rabbit anti-GFP and mouse monoclonal anti-Grt1 antibodies. (F) The Fiji-JACoP plugin was used to calculate the overlap (expressed as the Manders’ coefficient) between Sor4p and Grt1p for a field-of-view from ten cells as in E. The error bars show the s.d. The difference in cell size between samples is due to variable flattening by the coverslips. *P<0.0001 (two-tailed unpaired Student's *t*-test). Scale bars: 10 μm. DIC, differential interference contrast.

To test whether V-ATPase-a1p is required for heterotypic vesicle fusion, we used indirect immunofluorescence to localize Grt1p, Grl1p and Stx7l1p in fixed cells. In wild-type cells, Grl1p and Grt1p accumulate exclusively in docked mature mucocysts (Fig. 7A top). In contrast, in *Δv-atpase-a1* cells, both cargo proteins accumulate in a heterogeneous cohort of cytoplasmic vesicles (Fig. 7A middle). Compared to their localization in wild-type cells, Grl1p and Grt1p show less overlap (∼51%) in *Δv-atpase-a1* (Fig. 7B), which is similar to their overlap in *Δvps8a cells* (Fig. 7A bottom and Fig. 7B).

Consistent with previous results in wild-type cells (Kaur et al., 2017), Stx7l1p–GFP colocalized with Grt1p in docked mucocysts (Fig. 7C top; Fig. S6A). Their colocalization was ∼68% reduced in *Δv-atpase-a1* cells (Fig. 7C,D). Notably, a greater extent of colocalization was observed in the mutant cells between Grl3p and Stx7l1p (Fig. S6B,C).

However, since a large fraction of Stx7l1p appears as a diffuse cytoplasmic signal in Δ*v-atpase-a1* cells, the true compartmental colocalization of Stx7l1p and Grl3p is unknown. These results are consistent with previous reports that Stx7l1p is associated with vesicles containing Grl3p rather than Grt1p (Kaur et al., 2017; Sparvoli et al., 2018). Our results suggest that the absence of V-ATPase-a1 inhibits heterotypic fusion between Grl- and Grt-containing vesicles.

We previously found evidence that the targeting of Grt1p to mucocysts depends on its interaction with a sortilin/VPS10-family receptor, Sor4p (Briguglio et al., 2013). The Grt1p-containing vesicles in Δ*v-atpase-a1* cells, if they are stalled trafficking intermediates, might therefore also contain Sor4p. In agreement with this model, we found that the colocalization of Grt1p and Sor4p increased 3.5-fold in the Δ*v-atpase-a1* mutant compared to in wild-type cells (Fig. 7E,F).

**Fig. 8.**
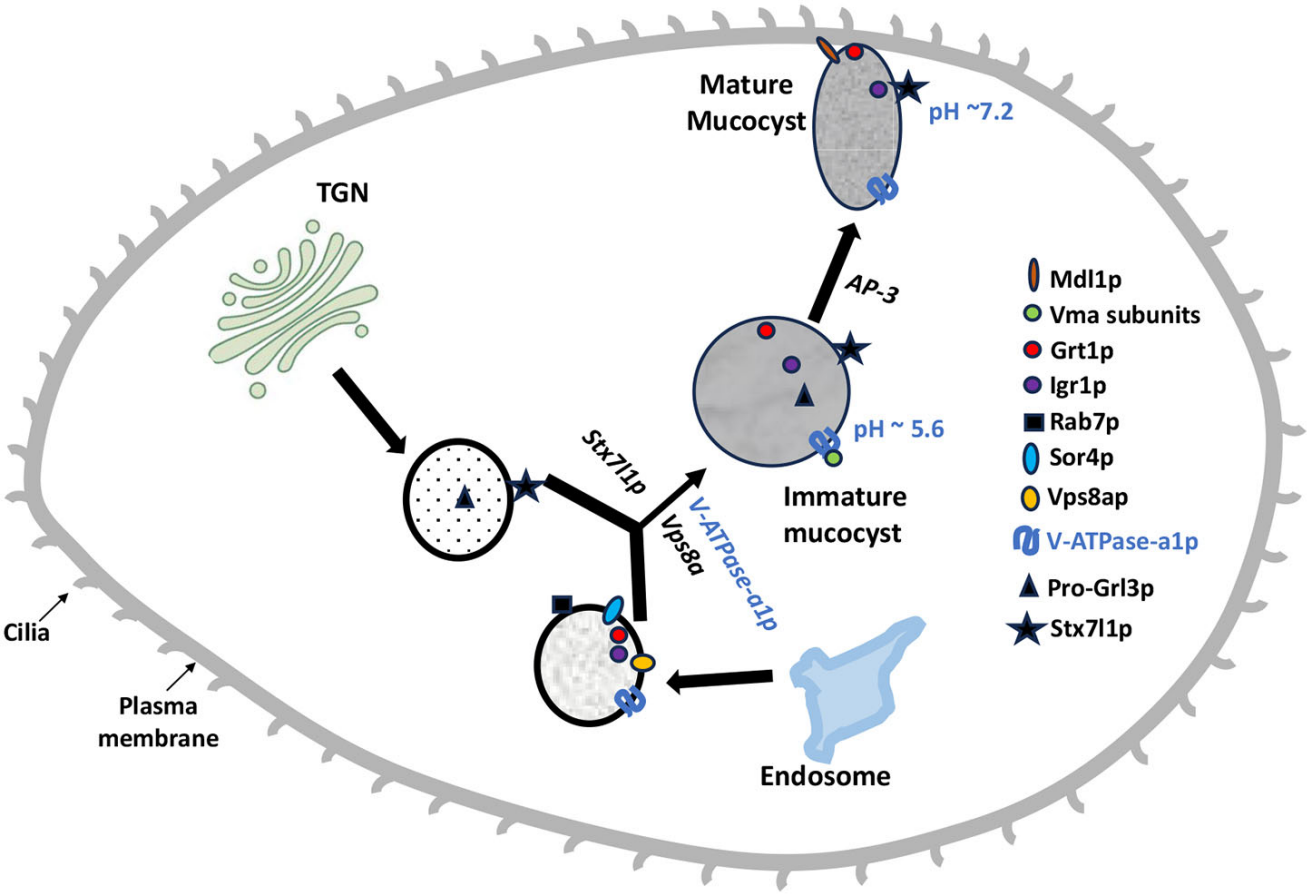
**Proposed model for the mucocyst biogenesis pathway in *T. thermophila*, extending that proposed by Sparvoli et al. (2018)**. Vps8ap and Stx7l1p are required for homotypic and/or heterotypic fusion of vesicles bearing Grl proproteins and other mucocyst cargo proteins, to generate immature mucocysts whose subsequent maturation depends on processing enzymes and the AP-3 adaptor. We now include V-ATPase-a1p in this model, which appears to be required for the same step of efficient vesicle fusion to form immature mucocysts. These are transiently acidified, and subsequently Vma subunits are lost via V-ATPase complex disassembly during mucocyst maturation. At the end of this process, the mature docked mucocysts are non-acidic.

To ask whether V-ATPase activity is required for heterotypic vesicle fusion, we treated wild-type cells with BafA1 for 16 h and used indirect immunofluorescence to identify Grt1p and Grl1p in fixed cells. After the BafA1 treatment, there was an increased accumulation of Grt1p and Grl1p within a heterogeneous cohort of cytoplasmic vesicles, as compared to control cells (Fig. S6D,E). Taken together, our results support the idea that V-ATPase-a1p promotes fusion of Grl1p-containing and Sor4p/Grt1-containing vesicles during mucocyst formation, and that the absence of V-ATPase-a1p results in phenotypes similar to those seen in *Δvps8a* cells. Based on these data, we proposed a model for the mucocyst biogenesis pathway in *T. thermophila* (Fig. 8), extending the model proposed by Sparvoli et al. (2018).

## DISCUSSION

In *T. thermophila*, the biogenesis of secretory vesicles known as mucocysts has previously been shown to depend on a set of proteins associated with LROs in other lineages (Kaur et al., 2017; Sparvoli et al., 2018). Here, we investigated whether a V-ATPase was also required for mucocyst formation. Based on genome annotation, *T. thermophila* expresses a large number of distinct V-ATPase subunits, many of which are likely to be compartment specific. Among the six *T. thermophila* paralogs encoding a-subunits of the V_0_ subcomplex, we discovered that the a1-subunit paralog is transcriptionally coregulated with known mucocyst-associated genes, and that the corresponding protein localizes strongly, though not exclusively, to mature docked mucocysts. In other organisms, the localization specificity of V-ATPases has been shown to depend on determinants in the a-subunit of the V_0_ subcomplex (Capecci and Forgac, 2013; Chen et al., 2024; Matsumoto et al., 2018; Oka et al., 2001; Sun-Wada and Wada, 2013, 2022; Sun-Wada et al., 2007; Toei et al., 2010). Consistent with this idea, in animal cells distinct a-subunits are found respectively in coated vesicles, the Golgi and/or early endosomes, and late endosomes and/or lysosomes (Breton and Brown, 2013; Chu et al., 2021; Matsumoto et al., 2018; Toei et al., 2010). The six genes encoding a-subunits of the V-ATPase in *T. thermophila* compare to just four a-subunit genes in mammals (Sun-Wada and Wada, 2013; Toei et al., 2010), whereas 17 a-subunit genes are present in *P. tetraurelia* (Wassmer et al., 2006). In *Paramecium,* these seventeen genes fall into nine families and localize to at least seven different compartments.

Notwithstanding the localization of a V-ATPase subunit to mucocysts, we demonstrate that they are similar to neutral pH storage LROs that have been documented in mammals, Amoebozoa and other lineages. That is, we found by three different approaches that mature mucocysts, which dock at the plasma membrane, are not detectibly acidic. The most impactful of these approaches was based on tagging the mucocyst cargo protein Grl3p with pHluorin, a fluorophore whose emission is pH sensitive. Using cells expressing this construct, we could also analyze biosynthetic mucocyst intermediates, namely cytoplasmic vesicles containing the mucocyst cargo protein Grl3p. Many of these vesicles were acidified, in contrast with mature mucocysts. Moreover, we directly measured the pH of mature and immature mucocysts, and our findings showed that the pH of mature mucocysts was non-acidic, whereas immature mucocysts showed an acidic pH. Thus, our data strongly suggest that mucocysts are transiently acidified during their biogenesis. We found that the a1-subunit gene is non-essential for cell viability but is required for mucocyst biogenesis. Similarly, the a3 subunit in *Paramecium* has been implicated in the formation of trichocysts (Wassmer et al., 2006). The transient acidification of ciliate secretory LROs at an intermediate phase in organellar maturation might be sufficient to explain the defects seen with knockout or silencing of the V-ATPase complex subunit genes. At the same time, it remains possible that specific subunits play roles distinct from proton pumping.

In *Tetrahymena* we detected numerous defects in mucocyst formation in *V-ATPase-a1*-knockout cells, including failure to process Grl proproteins as well as likely defects in heterotypic vesicle fusion that is a normal feature of mucocyst biogenesis. The proprotein processing defects are similar to those we previously found in cells lacking the gene encoding the key processing enzyme in mucocysts, *CTH3* (Kumar et al., 2014). This similarity in defects can be explained if transient acidification of the immature mucocyst is required to activate Cth3p. Similarly, the apparent defects in vesicle fusion, which resemble those in cells lacking the Vps8a subunit of a mucocyst-specific CORVET complex, could be indirect consequences of a failure to acidify one or more intermediates. Our data are consistent with this idea, since inhibition of acidification using BafA1 treatment produced defects similar to knockout of *V-ATPase-a1*, but further investigation will be needed to settle this issue (Coonrod et al., 2013; El Far and Seagar, 2011; Sreelatha et al., 2015; Strasser et al., 2011; Wang and Hiesinger, 2013). Interestingly, in mammalian cells there is evidence that the V-ATPase on secretory vesicles acts to acidify the lumen but also to regulate exocytosis machinery by acting as a pH sensor (Poëa-Guyon et al., 2013). The issue of potential subunit activities that are independent of proton pumping is also relevant for subunits like V-ATPase-a1 that remain mucocyst-associated after the holo-complex has disassembled during maturation.

The physiological regulation of V-ATPase in other organisms involves its reversible assembly and disassembly, among other mechanisms. For example, in yeast and mammalian cells, such reversible assembly of the V_1_ and V_0_ domains regulates endolysosomal and lysosomal pH (Lafourcade et al., 2008; Sava et al., 2024). Our results suggest that a similar phenomenon underlies the properties of mucocysts in *Tetrahymena*, unfolding in the context of an organellar maturation pathway. We found that the V_1_ subunits Vma1p, Vma8p and Vma10p are not present in mature, docked mucocysts, whereas they instead are found in a pool of cytoplasmic Grl3p-positive puncta that are likely to represent intermediates in mucocyst formation and/or maturation. These results suggest that the mucocyst-associated V-ATPase complex partially disassembles during maturation, with only a subset of the subunits remaining associated with the mucocyst . Importantly, at least one of the dissociated subunits, Vma8, has previously been shown to be essential for coupling of proton transport and ATP hydrolysis in yeast (Graham et al., 1995; Xu and Forgac, 2000). Therefore, the remnant V-ATPase complex in mature mucocysts is likely to be disabled for proton translocation, which can explain why mucocysts do not maintain an acidic luminal environment. Because non-acidic storage LROs are not unique to ciliates, similar phenomena could be an important feature of LRO specialization in other lineages.

## MATERIALS AND METHODS

### Reagents, *Tetrahymena* strains and culture conditions

*Tetrahymena thermophila* strains were grown overnight at 30°C with agitation in nutrient-rich SPP medium [2% proteose peptone (Gibco, USA), 0.2% dextrose (HiMedia, India), 0.1% yeast extract (Gibco, USA), 0.003% ferric EDTA (SRL, India)] supplemented with 250 μg/ml streptomycin sulfate, 250 μg/ml penicillin G and 0.25 μg/ml amphotericin B fungizone, to medium density (2–3×10^5^ cells/ml). Cu428 wild-type strain was used as a control for all experiments and for biolistic transformation. 10 mM Tris-HCl buffer (pH 7.4) was used for washing cells and as a starvation medium to reduce autofluorescence in food vacuoles. Cell culture densities were measured using a hemocytometer. *T. thermophila* strains used in this study are listed in Table S2. All reagents, chemicals, plasmids and other information about key resources are listed in Table S3. The constructs p2×HA-3×mCherry-Rab7p-NCVB, pGrl3p-GFP-CHX, pSor4p-GFP-CHX, p-2×mNeon-6c-myc-Neo4, p3×mCherry-2×HA-Neo4 and *Δvps8a*-Neo4 were from Sparvoli et al. (2018); pmEGFP-Neo4 and pNeo4 KO vector were from Kumar et al. (2014); pStx7l1p-GFP-Blasticidin was from Kaur et al. (2017). Plasmids pPOC1-mCherry-BSR and pPur4 were generously provided by Professor Chad Pearson (University of Colorado, USA) and Dr Kazufumi Mochizuki (University of Montpellier, France), respectively. Wild-type strain Cu428 was from Dr Abdur Rahaman (NISER, India). All experiments were performed three times unless otherwise indicated. The number of cells (*n*) analyzed for each experiment is represented in the corresponding figure. The *P*-value was determined via an unpaired Student's *t*-test (two-tailed) using MedCalc statistical software.

### Verification of knockout of genes by RT-PCR

RT-PCR was performed as described previously (Kumar et al., 2014). Briefly, total RNA was extracted by following the manufacturer's instructions using RNeasy mini spin column kits (Qiagen, USA). cDNA (200–500 base pairs of each gene) was synthesized and further amplified using the one step RT-PCR kit (Qiagen, USA) as per manufacturer's protocol, using primers designated in Table S4. To provide positive controls, RT-PCR using *β-tubulin*-specific primers was performed in parallel.

### Biolistic transformation

Biolistic transformations were carried out as described previously (Kumar et al., 2014, 2015) with some modifications. Briefly, cells were grown in SPP medium and incubated at 30^°^C with swirling to reach medium cell density. The next day, cells were washed twice with 10 mM Tris-HCl pH 7.4 and starved in the same buffer for 16–18 h. 15 µg of total linearized plasmid DNA was conjugated with 0.6 µm gold particles (Bio-Rad, USA) as per the manufacturer's instructions. At 4 h after bombardment, drugs were added to cultures that were swirling at 30°C to select positive transformants. Positive transformants were identified after 3–4 days and serially transferred 5 days/week in increasing concentrations of drug and decreasing concentrations of CdCl_2_ (up to 6.5 mg/ml of paromomycin, 0.1 µg/ml CdCl_2_; up to 200 µg/ml of puromycin and 0.5 µg/ml CdCl_2_; up to 60 µg/ml of blasticidin and 0.5 µg/ml CdCl_2_, 20 µg/ml of cycloheximide and 0.8 µg/ml CdCl_2_) for 4–5 weeks before further testing. Each line was evaluated using at least three independent transformants.

### Generation of *V-ATPase-a1* knockout strain

Upstream (∼1 kb) and downstream (∼1 kb) regions of the *V-ATPase-a1* (TTHERM_01332070) gene were amplified by PCR and subcloned into the SacI and XhoI sites of the pNeo4 vector, respectively, using the In-Fusion cloning kit (TaKaRa, Japan). *V-ATPase-a1* knockout construct was linearized using KpnI and SapI enzymes before biolistic transformation into Cu428 cells. The entire sequence of the *V-ATPase-a1* gene was deleted. The primers used to amplify target regions are listed in Table S4.

### Live-cell microscopy

Cultures for live microscopy were analyzed at mid-density (2–3×10^5^ cells/ml) unless otherwise indicated. To image cells expressing mNeonGreen-, mCherry-, GFP- or pHluorin-tagged fusion proteins, transformants were cultured overnight in SPP medium and then transferred into 10 mM Tris-HCl pH 7.4 for 2–4 h to reduce autofluorescence in food vacuoles. Colocalization in live cells was determined by simultaneous imaging of mNeonGreen or GFP-tagged fusion proteins with mCherry-tagged mucocyst markers. To simultaneously localize mNeonGreen-tagged V-ATPase-a1p with Lysotracker Red (Invitrogen, USA), cells were incubated with 200 nM Lysotracker Red for 5 min and images were captured as previously described (Kumar et al., 2014). For colocalization of V-ATPase-a1p and FM4-64 (Invitrogen, USA), cells were treated with 5 µM FM4-64, which labels endosomes, for 5 min and then pelleted and resuspended in 10 mM Tris-HCl pH 7.4. Subsequently, cells were imaged within 30–40 min. Cells expressing Grl3p–3×mCherry were incubated with 0.1 µg/ml Acridine Orange (Invitrogen, USA) for 2 min and 5 µM Protonex Green (AAT Bioquest, USA) for 120 min at room temperature and in the dark. Cells were washed twice with 10 mM Tris-HCl pH 7.4 and observed under the microscope. For bafilomycin A1 (BafA1) treatment, a mid-density culture was starved in 10 mM Tris-HCl pH 7.4 with 1% DMSO containing 1 µM BafA1 for 16 h. Cells treated with only 1% DMSO were used as the control. To check colocalization between V-ATPase-a1p and Rab7 positive endosomes, cells were transformed to co-express V-ATPAse-a1p at the endogenous locus with mCherry–Rab7p. Transgene mCherry–Rab7p was induced for 2 h in SPP with 2 µg/ml CdCl_2_ followed by induction with 0.5 μg/ml CdCl_2_ for 2 h in starvation buffer 10 mM Tris-HCl, pH 7.4. Stx7l1p–GFP was induced with CdCl_2_ as mentioned above. Live-cell images were captured using an Olympus SR10 spinning disk super-resolution confocal microscope (Olympus, Japan). Both confocal and super-resolution modes with a 60× oil objective were used to capture time-lapse videos. For *z* sectioning, SORA disk-enabled acquisition was used to keep the *z* slice step size at 0.3 µm. After removing the background signal from the images, they were saved as JPEGs that were denoised, colored, and adjusted for brightness and contrast using the Fiji application (http://fiji.sc/Fiji). Colocalization was measured by the Fiji BIOP JACoP plugin and fluorescence intensity line profile plot using the Fiji application.

### pH measurement

Grl3p–pHluorin–mCherry-expressing cells were starved in DMC pH 7 (Haddad and Turkewitz, 1997) with 100 mM KCl for 2 h. Live images were captured within 45 min in both the green and red channel, maintaining the same exposure and laser power on an Olympus SR10 spinning disk super-resolution confocal microscope with a 60× oil objective.

To generate a standard curve for pH measurement (https://documents.thermofisher.com/TFS-Assets/LSG/manuals/phi_calibration_buffer_kit_qrc.pdf), after starvation, cells were treated with ionophores (10 µM nigericin and 10 µM valinomycin) in different pH buffers (pH 4.7, 5.5, 6.0, 6.5, 7.0 and 7.5) for 5 min and live images were captured as mentioned above. The images were processed to adjust brightness and contrast using auto mode then converted into 8-bit images, thresholds were adjusted in the green and red channels, noise was removed, and MFI for both pHluorin and mCherry was measured using the Fiji application. The ratio of MFI of mCherry to pHluorin was measured at various pH levels, and the data were plotted using a scatter plot with a fit linear option using Origin software version 5.0.

### Immunofluorescence assay

Cells were grown to reach a cell density of 5×10^5^ cells/ml, then starved in 10 mM Tris-HCl pH 7.4 for 2 h, fixed, and immunolabeled as described previously (Kumar et al., 2014). Monoclonal antibodies 5E9 (1:9; Bowman and Turkewitz, 2001) and 4D11 (1:5; Turkewitz and Kelly, 1992) were used to visualize Grl3p and Grt1p, respectively followed by goat anti-mouse IgG conjugated with Texas Red (1:100; Invitrogen, USA). Grl1p and GFP-tagged fusion proteins were visualized by 1:5000-diluted rabbit anti-Grl1 (Kuppannan et al., 2022; Ota, 2018) and rabbit anti-GFP (1:400; Invitrogen, USA), respectively, followed by anti-rabbit IgG antibody conjugated with Alexa Fluor 488 (1:250; Invitrogen, USA). Cells were double immunolabeled with mouse mAb 4D11 and rabbit anti-Grl1 to visualize the fusion of Grl1p- and Grt1p-containing vesicles. Fixed cells were imaged on a Leica SP5 II STED-CW super-resolution laser scanning confocal microscope (Leica, Germany) or an Olympus SR10 spinning disk super-resolution confocal microscope with a 60× oil objective. Images were analyzed as described above.

### Electron microscopy

Cells were grown to reach high cell density (10^6^/ml), washed with 10 mM Tris-HCl (pH 7.4), fixed, section stained and imaged as described previously (Kumar et al., 2015).

### SDS-PAGE and western blotting

Whole-cell lysates were prepared from 3×10^5^ cells as described previously (Kumar et al., 2014). Using mouse anti-c-Myc agarose beads (Thermo Fisher Scientific, USA), 2×mNeon-6c-myc-tagged fusion proteins were immunoprecipitated from detergent lysates as previously described (Sparvoli et al., 2018). Samples were separated using SDS-PAGE and then transferred to 0.45 μm PVDF membranes (Merck Millipore, USA) for western blots. Blots were blocked and probed with antibodies described previously (Turkewitz et al., 1991). The primary antibodies rabbit anti-Grl1, rabbit anti-Grl3 (Kumar et al., 2014, 2015), mouse monoclonal anti-GFP (Biolegend, USA) and anti-c-Myc (Sigma, USA) were diluted 1:2000, 1:800, 1:5000 and 1:4000, respectively. Proteins were visualized using Super Signal West Femto Maximum Sensitivity Substrate (Thermo Fisher Scientific, USA), and enhanced chemiluminescence horseradish peroxidase (HRP)-linked anti-rabbit IgG (Bio-Rad, USA), anti-mouse IgG (Bio-Rad, USA) and anti-mouse IgG for immunoprecipitation (Abcam, UK), with the secondary antibodies diluted 1:5000, 1:5000 and 1:1000, respectively.

### Expression of V-ATPase-a1p, V-ATPase-a2p, Vma1p, Vma8p, Vma10p, Cth3p and Grl3p at endogenous locus

The p2×mNeon-6c-myc-Neo4 and pmEGFP-Neo4 vectors were previously described (Briguglio et al., 2013; Kumar et al., 2014; Sparvoli et al., 2018). To create pV-ATPase-a1p-2×mNeon-6c-myc-Neo4, the genomic DNA of V-*ATPase*-a1 (TTHERM_01332070; 1462 bp) without stop codon and 1000 bp of V-*ATPase*-a1 downstream genomic region were amplified and cloned into p2×mNeon-6c-myc-Neo4 vector at NotI and XhoI sites, respectively, using In-Fusion Cloning (TaKaRa, Japan). The construct was named pV-ATPase-a1p-2×mNeon-6c-myc-Neo4 and was linearized with KpnI and SacI, 2×mNeon-6c-myc was fused to the C terminus of V-*ATPase*-a1 at the endogenous macronuclear locus via homologous recombination. A similar approach was used to amplify the genomic DNA without stop codon (∼700–900 bp) and downstream genomic sequence (∼600–850 bp) of other V-ATPase subunits – *V-ATPase-a2* (TTHERM_00463420), *VMA1* (TTHERM_00339640), *VMA8* (TTHERM_00821870) and *VMA10* (TTHERM_00052460) – for fusion of 2×mNeon-6c-myc to the C terminus of V-ATPase subunits at the endogenous macronuclear locus. The same approach was used to clone *VMA1* in the p3×mCherry-2HA-Pur4 vector. For the generation of pGrl3p-3×mCherry-2xHA-Pur4, the pGrl3p-GFP-CHX vector was used as template (Sparvoli et al., 2018). BamHI/SpeI sites were used to replace monomeric mGFP with p3×mCherry-2xHA, while PstI/HindIII sites were used to replace CHX (cycloheximide resistance gene) with Pur4 (puromycin resistance gene) to generate pGrl3p-3×mCherry-2×HA-Pur4 vector. The pmEGFP-Neo4 and pGrl3p-3×mCherry-2×HA-Pur4 vectors were used to make the pGrl3p-mEGFP-Neo4 construct. The genomic (857 bp excluding stop codon) and downstream sequence (783 bp) of *GRL3* gene were excised from pGrl3p-3×mCherry-Pur4 vector using BamHI/SacI and HindIII/KpnI, respectively, and ligated into pmEGFP-Neo4 vector at mentioned sites, respectively, using T4 DNA ligase (New England, Biolabs, USA). pGrl3p-mEGFP-Neo4 vector was used as template to generate pGrl3p-pHluorin-Neo4 construct. Super-ecliptic pHluorin (SEP) variant sequence was derived from the pHluorin MT1-MMP vector (Lizárraga et al., 2009). The pHluorin gene (lacking stop codon), codon optimized for *Tetrahymena* using the IDT Codon optimization tool, was synthesized (Eurofins Genomics Pvt Ltd) with added BamHI/SpeI sites at 5′ and 3′ ends of the gene. Monomeric mGFP from pGrl3p-mEGFP-Neo4 was replaced with pHluorin using BamHI/SpeI sites to generate pGrl3p-pHluorin-Neo4. To construct pGrl3-pHluorin-mCherry-Pur4, the Neo4 cassette of pGrl3p-pHluorin-Neo4 was replaced by Pur4 from pGrl3-3×mCherry-Pur4 using Kpn1 and Pst1 sites to generate pGrl3-pHluorin-Pur4. The mCherry gene fragment was amplified from pPOC1-mCherry-BSR using primers designated in Table S4 and cloned into pGrl3-pHluorin-Pur4 vector at the SpeI site using In-fusion cloning. All constructs were confirmed by DNA sequencing. The final constructs were linearized with SacI and KpnI before transformation. The p2×HA-3×mCherry-Rab7p-Ncvb construct was linearized by digestion with SfiI and transformed into cells expressing V-ATPase-a1p by biolistic transformation. pGrl3p-3×mCherry-2×HA-Pur4, pGrl3p-mEGFP-Neo4 and pGrl3p-pHluorin-Neo4 were transformed into wild-type Cu428 cells using biolistic transformation. pGrl3-pHluorin-mCherry-Pur4 vector was transformed into Cu428 and *MN173* cells. V-ATPase-a1p-2×mNeon-6c-myc-Neo4, pV-ATPase-a2p-2×mNeon-6c-myc-Neo4, pVma1p-2×mNeon-6c-myc-Neo4, pVma8p-2×mNeon-6c-myc-Neo4 and pVma10p-2×mNeon-6c-myc-Neo4 vectors were transformed into Cu428 cells expressing Grl3p-3×mCherry-2×HA-Pur4. pVma1p-3×mCherry-2×HA-Pur4 vector was transformed into Cu428 cells expressing V-ATPase-a1p-2×mNeon-6c-myc-Neo4.

### *In silico* analyses

Protein sequences of V-ATPase-a subunit paralogs (a1p–a6p) of *T. thermophila* were downloaded from the TGD website (https://tet.ciliate.org/) and combined to create a FASTA file for multiple sequence alignment. The alignment was conducted using the Clustal X2 software, with Clustal as the output format. The aligned file was opened in Jalview (https://www.jalview.org/), a visualization software, to measure sequence homology. The sequence homology was also analyzed by EMBOSS Needle (https://www.ebi.ac.uk/jdispatcher/psa/emboss_needle) using the default parameters. Transcription profiles were downloaded from the Tetrahymena Functional Genomics Database (http://tfgd.ihb.ac.cn; Table S5) (Miao et al., 2009; Xiong et al., 2011, 2013). For plotting, each profile was normalized by setting the maximum expression level of the gene to 1. The V-ATPase-a1p structure was predicted using Protein Homology/Analogy Recognition Engine V 2.0 (Phyre 2), a web portal for protein modeling (Kelley et al., 2015). Structural homologs of the *T. thermophila* V-ATPase-a1p were identified using the AlphaFold Protein Structure Database (https://alphafold.ebi.ac.uk/).

## Supplementary Material

10.1242/joces.264146_sup1 Supplementary information
