# Supplementary material for: Maturation stage-specific V-ATPase disassembly explains the neutral pH of mature mucocyst lysosome-related organelles in Tetrahymena thermophila
Source: J Cell Sci. 2025 Dec 9;138(23):jcs264146. doi: 10.1242/jcs.264146 (PMC12752500; doi:10.1242/jcs.264146)
Supplement: Supplementary information [file joces-138-264146-s1.pdf]

A

FigS1

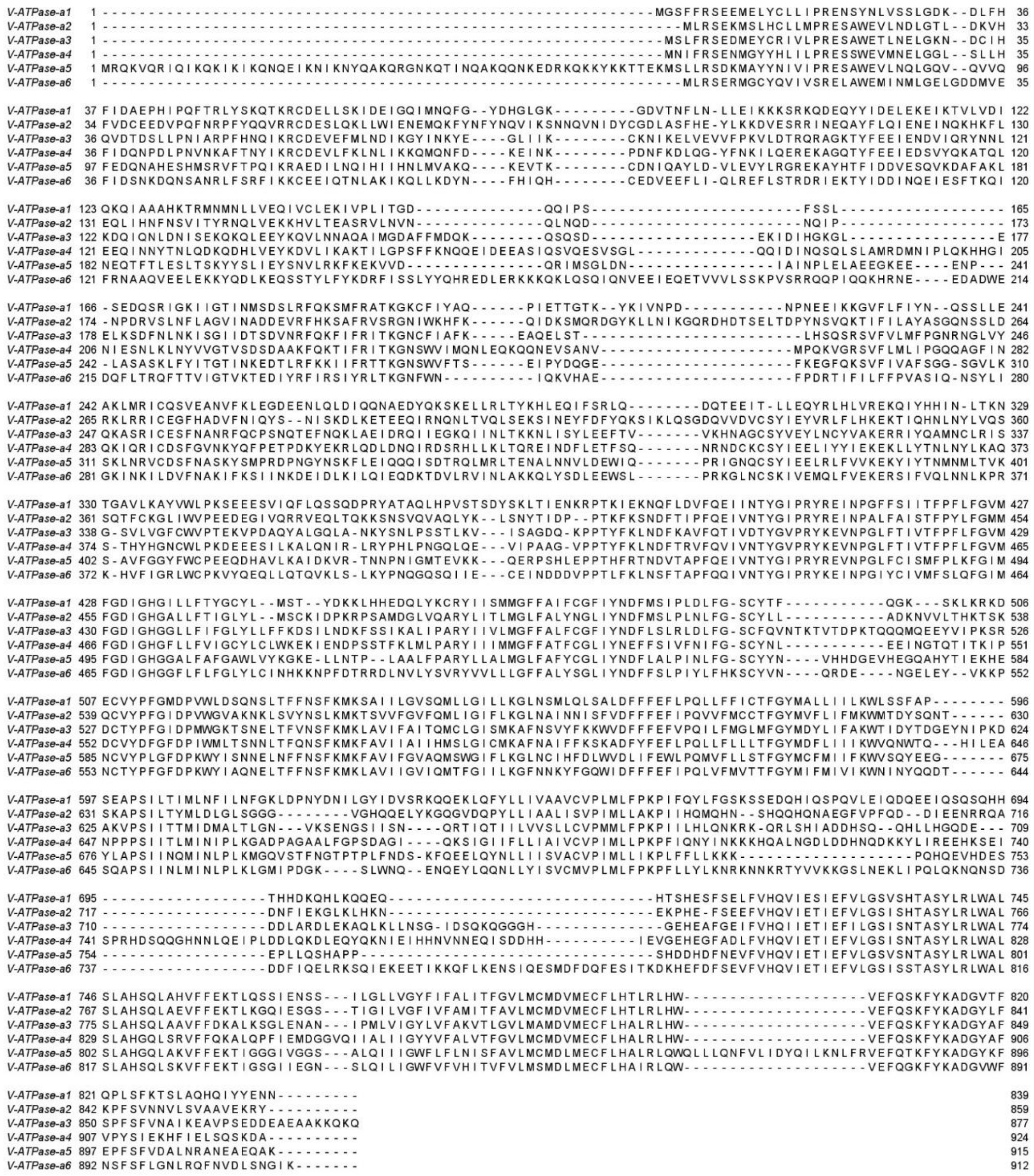

B **The amino acid identity between different V-ATPase-a subunits of Tetrahymena**

	V-ATPase-a1	V-ATPase-a2	V-ATPase-a3	V-ATPase-a4	V-ATPase-a5	V-ATPase-a6
**V-ATPase-a1**	100%	41.98%	37.53%	37.57%	36.26%	35.55%
**V-ATPase-a2**	41.98%	100%	38.30%	36.10%	36.10%	36.10%
**V-ATPase-a3**	37.53%	38.30%	100%	44.80%	36.40%	36.80%
**V-ATPase-a4**	37.57%	36.10%	44.80%	100%	36.80%	36.60%
**V-ATPase-a5**	36.26%	36.10%	36.40%	36.80%	100%	36.70%
**V-ATPase-a6**	35.55%	36.10%	36.80%	36.60%	36.70%	100%

Fig. S1. (A) The sequence alignment of *Tetrahymena* V-ATPase subunit-a paralogs. (B) Percent identity of protein sequence of *Tetrahymena* V-ATPase a-subunit paralogs.

Journal of Cell Science • Supplementary information

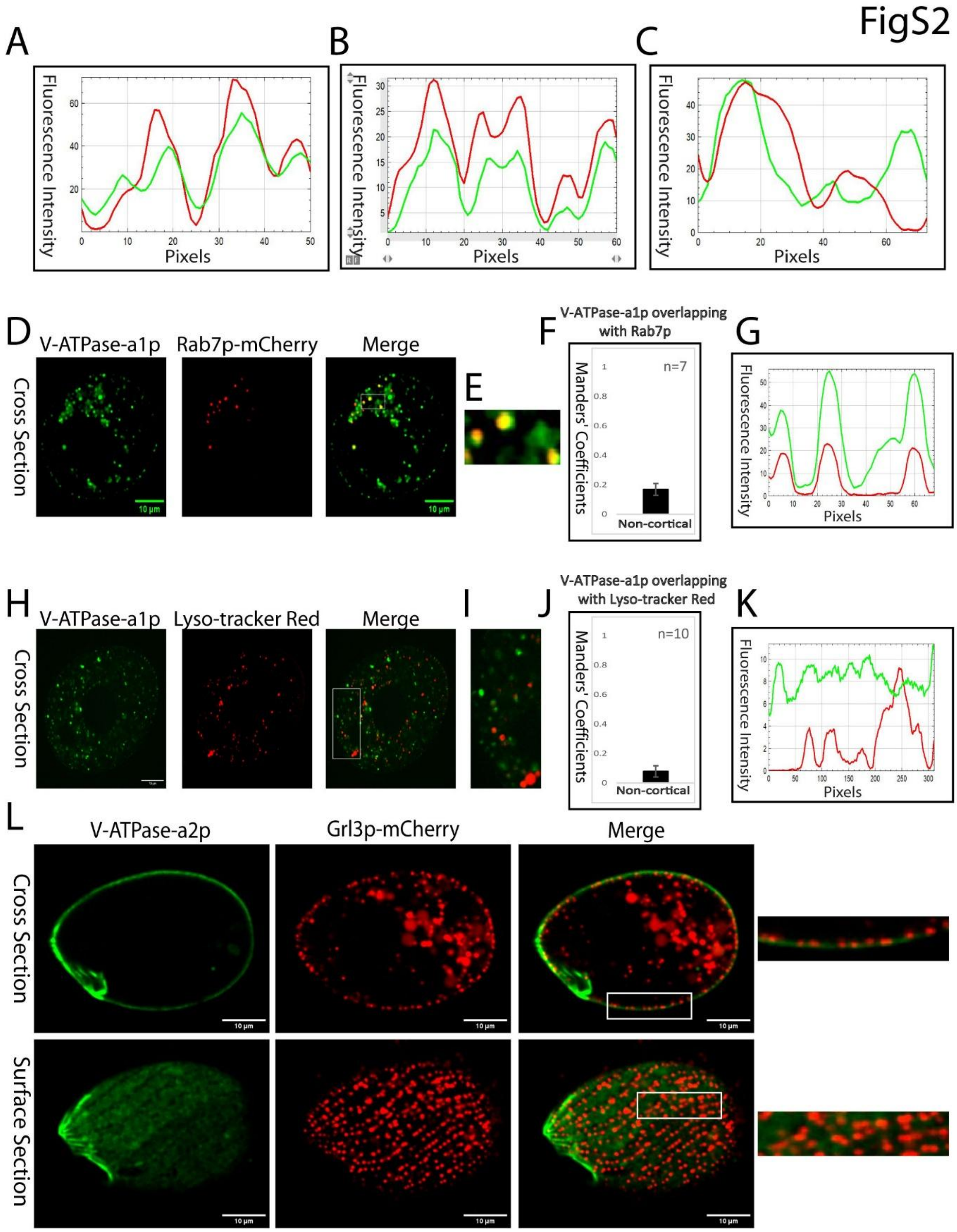

Fig. S2. (A-C) Related to Figure 2B. V-ATPase-a1p and Grl3p fluorescence intensity graphs along the length of the rectangle shown in panel 2B insets a, b and c, respectively. (D) V-ATPase-a1p is co-localized with Rab7 positive endosomes. Cells were transformed to co-express V-ATPase-a1p at the endogenous locus with mCherry-Rab7p. Rab7 transgene was induced for 2h in SPP with 2 µg/ml $CdCl_2$, further induction with 0.5µg/ml $CdCl_2$ for 2hrs in starvation buffer (10 mM Tris, pH 7.4). (E) Panel E shows co-localizations between V-ATPase-a1p and Rab7p; the inset is from panel D. (F) 7 non-overlapping images from panels

Journal of Cell Science • Supplementary information

D was used to calculate the percentage of overlap (Mander's coefficient) between V-ATPase-a1p and Rab7p in non-cortical compartment. Co-localization was measured using the Fiji-BIOP JACoP plugin as before. (G) Fluorescence intensity graphs of V-ATPase-a1p and Rab7p are illustrated along the length of the rectangle as shown in panel S2D. (H) Measurement of overlap between non–cortical fraction of V-ATPase-a1p with Lyso-tracker Red. Cells expressing V-ATPase-a1p were incubated with 200 nM Lyso-tracker Red for 5 min and images were captured. (I) Panel I shows extent of co-localizations between V-ATPase-a1p and Lyso-tracker Red. (J) Co-localization was measured using the Fiji-BIOP JACoP plugin from 10 non-overlapping images from panels H as mentioned in panel F. (K) Fluorescence intensity graphs for V-ATPase-a1p and Lyso-tracker Red are presented along the length of the rectangle as shown in panel S2H. (L) Expression and localization of V-ATPase-a2p. In optical cell cross and surface sections (top and bottom, respectively), cells co-expressing endogenously 2xmNeon-tagged V-ATPase-a2p with 3xmCherry tagged Grl3p (mucocyst marker). V-ATPase-a2p is not associated with the docked mucocyst at the cell cortex or surface, as indicated in the selected rectangular region on the right. Scale bars, 10 μm.

Journal of Cell Science • Supplementary information

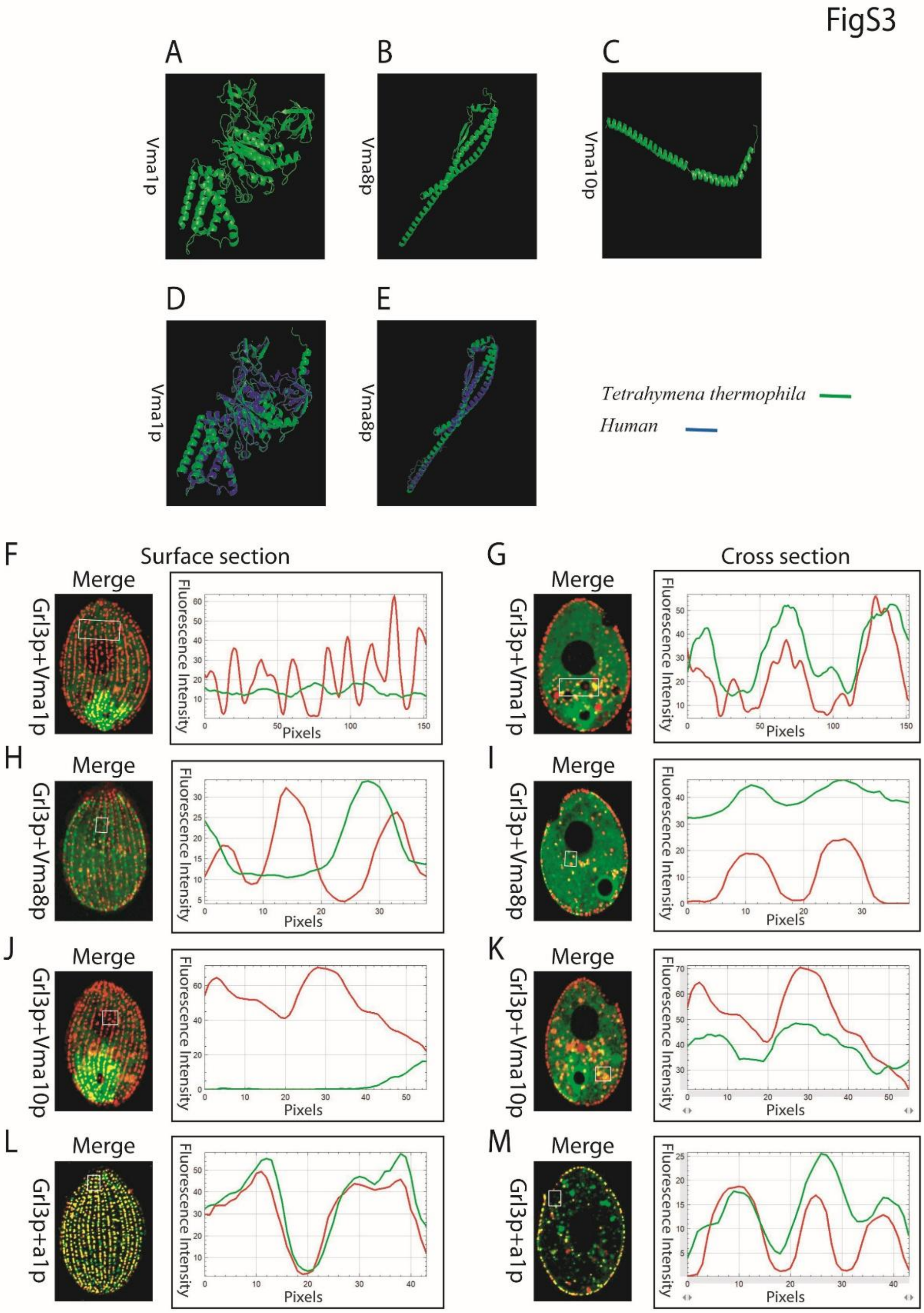

Fig. S3. (A-E) Related to **Figure 5.** (A) The Phyre2 predicted structure of *T. thermophila* Vma1p compared with subunit-A of the V_1 domain of mammalian cells showed 64% structural identity with 100% confidence, 97% coverage. (B) The predicted structure of Vma8p had 47% structural identity with subunit-D of the V_1 domain of mammalian cells with 100% confidence, 83% coverage. (C) *T. thermophila* Vma10p also showed 28% predicted structural similarity to a V-ATPase subunit in *S. cerevisiae*, subunit-G with 62% coverage. (D-E) The predicted structure of (D)Vma1p (A subunit of the V_1 domain of the V-ATPase complex) and (E) Vma8p (the D subunit of the V_1 domain of the V-ATPase complex) of *Tetrahymena thermophila* (green) and humans (blue) by AlphaFold structure prediction. Vma1p and Vma8p structures of *T. thermophila* and humans are superimposed using PyMOL software. (F-M) Related to panel 2C-H and 2K-L. Fluorescence intensity graphs of Grl3p and V-ATPase subunits are illustrated along the length of the rectangle as shown in panel S3F-S3M.

Journal of Cell Science • Supplementary information

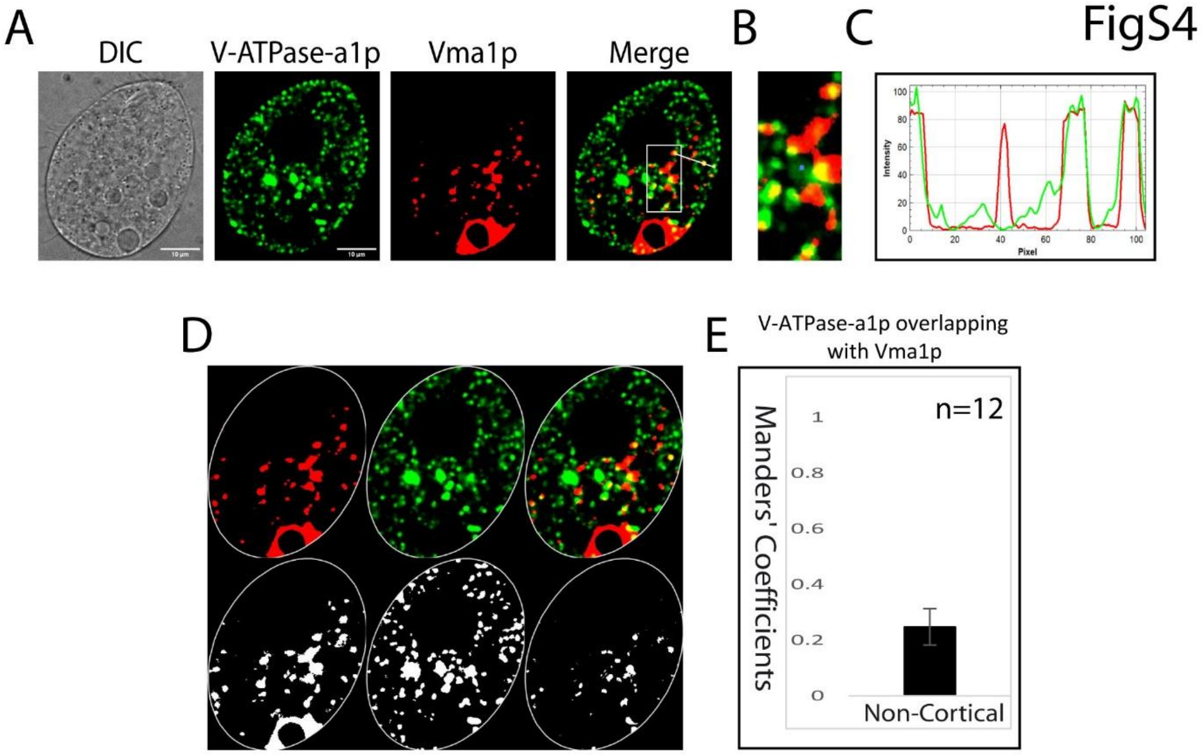

Fig. S4. (A-E) Overlap between Vma1p is with a1p subunit in non-cortical structures. (A) Cells co-expressing 2xmNeon-tagged a1p subunit with Vma1p-3xmCherry, both at their endogenous loci. (B) The inset from panel S4A shows co-localization of a1p with Vma1p. (C) Fluorescence intensity graphs for a1p and Vma1p are shown along the length of the rectangle depicted in panel S4A. (D) The cross sections (panel A) were used to measure colocalization in non-cortical structures. To measure co-localization, the images were processed to remove noise and adjust thresholds, with co-localization quantified using the Fiji-BIOP JACoP plugin as described. (E) Twelve non-overlapping images from panels D were used to calculate the percentage of overlap (Mander's coefficient) between a1p and Vma1p in non-cortical (cross section) regions. The error bars show the SDs.

Journal of Cell Science • Supplementary information

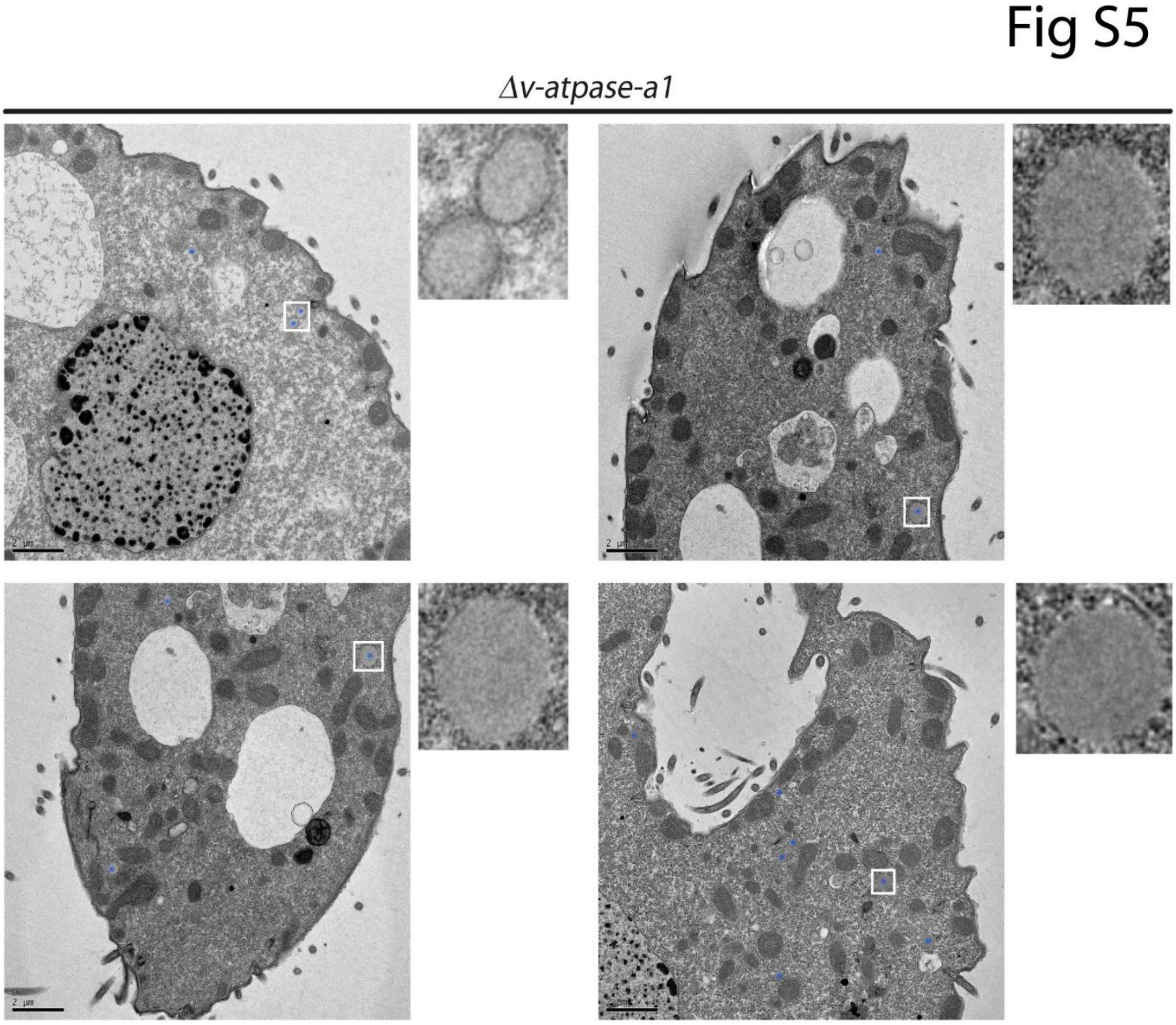

Fig. S5. Related to **Figure 6E**. Electron micrographs show mucocysts in *Δv-atpase-a1* cells. Only immature or intermediate mucocysts are seen in *Δv-atpase-a1* cells. The inset displays a magnified view of mucocysts. Scale bars, 2 µm.

Journal of Cell Science • Supplementary information

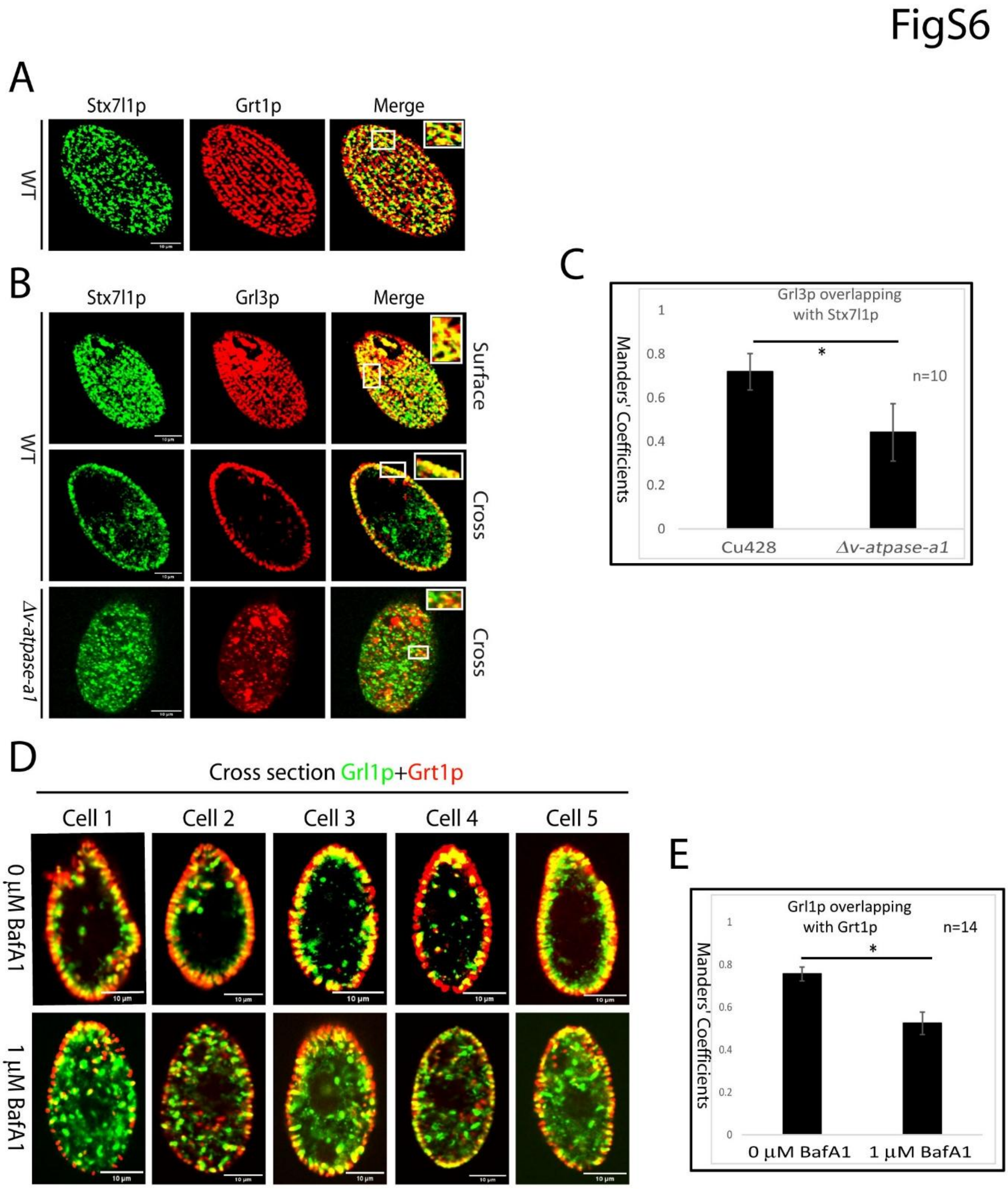

Fig. S6. (A) Surface section of the wild-type cell shown in **Figure 7C**. (B) Wild-type (top) and *Δv-atpase-a1* (bottom) cells expressing Stx7l1p-GFP were stained with rabbit anti-GFP and mouse monoclonal anti-Grl3 (5E9) antibodies. In *Δv-atpase-a1* cells, the large fraction of Grl3p co-localizes with Stx7l1p-GFP. The inset demonstrates the co-localization of Stx7l1p with Grl3p. (C) A sample of ten non-overlapping images was used to calculate the percentage of overlap (Mander's coefficient) between Stx7l1p and Grl3p using the Fiji-JACoP plugin, as previously described. (D) After starvation, WT cells were treated with 1% DMSO or 1% DMSO containing 1 μM BafA1 for 16 hrs, as previously described. The cells were double stained with rabbit anti-Grl1 and mouse monoclonal anti-Grt1 (4D11). A notable reduction in the co-localization of Grl1p and Grt1p was observed after BafA1 treatment. (E) A sample of 14 non-overlapping images was used to calculate the percentage of overlap (Mander's coefficient) between Grl1p and Grt1p using the Fiji-BIOP JACoP plugin as before. The error bars represent the standard deviations. Images depict cell cross sections with scale bars of 10μm. Asterisks (*) shows a p value of <0.0001.

Journal of Cell Science • Supplementary information

Table S1. Identification of *T. thermophila* V-ATPase subunits. Related to Fig. 4.

	Subunit[a]	Yeast Gene (Saccharomyces cerevisiae)	Tetrahymena ID	Proposed Name	E Value[b]	Query cover %	% Identity[c]
V_1 domain	A	*VMA1*	TTHERM_00339640	*VMA1*	0	98%	57.91%
	D	*VMA8*	TTHERM_00821870	*VMA8*	4e-45	80%	46.45%
	G	*VMA10*	TTHERM_00052460	*VMA10*	2e-04	95%	30.28%

a: Subunits of a V-ATPase.

b: Expectation value generated using DELTA-BLASTp analysis.

c: Percent identity of protein sequence of V-ATPase subunits compared with *S. cerevisiae*.

Table S2. *Tetrahymena* Strains used in this study

Strain name	Phenotype	Details of relevant genetic modification	Source
Cu428	Wild type	None	Dr Abdur Rahaman (NISER, India)
MN173	Exocytosis (exo-) mutant, the mucocysts fail to dock at the plasma membrane	Unknown	(Chilcoat et al., 1996; Melia et al., 1998)
SKBN001	*Δv-atpase-a1;* no detectible *V-ATPase-a1* expression	Replaces nucleotides 1 to 3479 bp of macronuclear *V-ATPase-a1* ORF with Neo4 cassette.	This study
SKBN002	Endogenous-level expression of 2xmNeon-6xc-myc-tagged V-ATPase-a1p	C-terminal fusion of V-ATPase-a1p and 2xmNeon-6xc-myc with Neo4 cassette, integrated at the macronuclear *V-ATPase-a1* locus	This study
SKBN003	Endogenous-level expression of 3xmCherry-2xHA-tagged Grl3p	C-terminal fusion of Grl3p and 3xmCherry-2xHA with Pur4 cassette, integrated at the macronuclear *GRL3* locus	This study
SKBN004	Endogenous-level expression of 2xmNeon-6xc-myc-tagged V-ATPase-a1p and 3xmCherry-2xHA-tagged Grl3p	C-terminal fusion of V-ATPase-a1p and 2xmNeon-6xc-myc with Neo4 cassette, integrated at the macronuclear *V-ATPase-a1* locus. Also, C-terminal fusion of Grl3p and 3xmCherry-2xHA with Pur4 cassette, integrated at the macronuclear *GRL3* locus	This study
SKBN005	Endogenous-level expression of 2xmNeon-6xc-myc-tagged V-ATPase-a1p. Inducible expression of 2xHA-3xmCherry-tagged Rab7p	C-terminal fusion of V-ATPase-a1p and 2xmNeon-6xc-myc with Neo4 cassette, integrated at the macronuclear *V-ATPase-a1* locus. Also, N-terminal fusion of Rab7p and 2xHA-3xmCherry, expressed under the control of the *MTT1* promoter, at the macronuclear *MTT1* locus.	This study
SKBN006	Endogenous-level expression of 2xmNeon-6xc-myc-tagged V-ATPase-a2p and 3xmCherry-2xHA-tagged Grl3p	C-terminal fusion of V-ATPase-a2p and 2xmNeon-6xc-myc with Neo4 cassette, integrated at the macronuclear *V-ATPase-a2* locus. Also, C-terminal fusion of Grl3p and 3xmCherry-2xHA with Pur4 cassette, integrated at the macronuclear *GRL3* locus	This study
SKBN007	Endogenous-level expression of GFP-tagged Grl3p	C-terminal fusion of Grl3p and GFP with Neo4 cassette, integrated at the macronuclear *GRL3* locus	This study
SKBN008	Endogenous-level expression of pHluorin-tagged Grl3p	C-terminal fusion of Grl3p and pHluorin with Neo4 cassette, integrated at the macronuclear *GRL3* locus	This study
SKBN009	Endogenous-level expression of GFP-tagged Sor4p.	C-terminal fusion of Sor4p and GFP with CHX cassette, integrated at the macronuclear *SOR4* locus.	This study
SKBN010	Endogenous-level expression of GFP-tagged Sor4p. *Δv-atpase-a1;* no detectible *V-ATPase-a1* expression.	C-terminal fusion of Sor4p and GFP with CHX cassette, integrated at the macronuclear *SOR4* locus. Replaces nucleotides 1 to 3479 bp of macronuclear V-ATPase-a1 ORF with Neo4 cassette.	This study
SKBN011	Endogenous-level expression of 2xmNeon-6xc-myc-tagged	C-terminal fusion of Vma1p and 2xmNeon-6xc-myc with Neo4 cassette, integrated at	This study

Journal of Cell Science • Supplementary information

	Vma1p and 3xmCherry-2xHA-tagged Grl3p	the macronuclear *VMA1* locus. Also, C-terminal fusion of Grl3p and 3xmCherry-2xHA with Pur4 cassette, integrated at the macronuclear *GRL3* locus	
SKBN012	Endogenous-level expression of 2xmNeon-6xc-myc-tagged Vma8p and 3xmCherry-2xHA-tagged Grl3p	C-terminal fusion of Vma8p and 2xmNeon-6xc-myc with Neo4 cassette, integrated at the macronuclear *VMA8* locus. Also, C-terminal fusion of Grl3p and 3xmCherry-2xHA with Pur4 cassette, integrated at the macronuclear *GRL3* locus	This study
SKBN013	Endogenous-level expression of 2xmNeon-6xc-myc-tagged Vma10p and 3xmCherry-2xHA-tagged Grl3p	C-terminal fusion of Vma10p and 2xmNeon-6xc-myc with Neo4 cassette, integrated at the macronuclear *VMA10* locus. Also, C-terminal fusion of Grl3p and 3xmCherry-2xHA with Pur4 cassette, integrated at the macronuclear *GRL3* locus	This study
SKBN014	Endogenous-level expression of pHluorin-mCherry tagged Grl3p	C-terminal fusion of Grl3p and pHluorin-mCherry with Pur4 cassette, integrated at the macronuclear *GRL3* locus of Cu428 cells	This study
SKBN015	Endogenous-level expression of pHluorin-mCherry tagged Grl3p.	C-terminal fusion of Grl3p and pHluorin-mCherry with Pur4 cassette, integrated at the macronuclear *GRL3* locus of MN173 cells	This study
SKBN016	Endogenous-level expression of 2xmNeon-6xc-myc-tagged V-ATPase-a1p and 3xmCherry-2xHA-tagged Vma1p	C-terminal fusion of V-ATPase-a1p and 2xmNeon-6xc-myc with Neo4 cassette, integrated at the macronuclear *V-ATPase-a1* locus. Also, C-terminal fusion of Vma1p and 3xmCherry-2xHA with Pur4 cassette, integrated at the macronuclear *VMA1* locus	This study
SKBN017	Inducible expression of GFP-tagged Stx7l1p.	C-terminal fusion of Stx7l1p and GFP with BLAS cassette, integrated at the macronuclear *MTT1* locus.	This study
SKBN018	Inducible expression of GFP-tagged Stx7l1p. *Δv-atpase-a1;* no detectible *V-ATPase-a1* expression.	C-terminal fusion of Xtx7l1p and GFP with BLAS cassette, integrated at the macronuclear *MTT1* locus. Replaces nucleotides 1 to 3479 bp of macronuclear V-ATPase-a1 ORF with Neo4 cassette.	This study

Table S3. Key Resources

Chemicals and Reagents		
**Chemicals and Reagents**	**Source**	**Catalog number**
Cadmium Chloride	Sigma Aldrich	239208-100G
Dibucaine hydrochloride	Sigma Aldrich	D0638-5G
Paromomycin sulphate salt	Sigma Aldrich	P5057-5G
Cycloheximide	Sigma Aldrich	C1988-1G
Valinomycin	Millipore Sigma	94675-10MG
Nigericin sodium salt	Millipore Sigma	N7143-5MG
Blasticidin	InvivoGen	ant-bl-05
Bafilomycin A1	InvivoGen	tlrl-baf1
FM™ 4-64	Invitrogen™	T3166
LysoTracker™ Red	Invitrogen™	L7528
Ampicillin	G Biosciences	AB1005
cOmplete, EDTA-free	Roche	11836170001
PMSF	SRL	87606
Dextrose anhydrous	HiMEDIA	TC130-100G
Proteose Peptone	GIBCO	211684
EDTA Ferric Monosodium salt	SRL	59389
Yeast Extract	GIBCO	212750
BSA	HiMEDIA	MB083-100G
Triton X-100	G Biosciences	RC1219
Tween® 20	G Biosciences	RC1226
Paraformaldehyde	Sigma Aldrich	16005
IGEPAL® CA-630 (NP40)	Sigma Aldrich	I3021-50ML

Journal of Cell Science • Supplementary information

SuperSignal™ West Femto Maximum Sensitivity Substrate	Thermo Scientific™	34094
Acridine Orange	Invitrogen™	A1301
Protonex™ Green 500	AAT Bioquest®	21215
Immobilon®-P PVDF Membrane	Merck Millipore	IPVH00010
RNeasy® Mini kit	Qiagen	74104
QIAGEN OneStep RT-PCR kit	Qiagen	210210
QIAprep® Spin Miniprep Kit	Qiagen	27104
0.6µm Gold Microcarrier	Bio-Rad	165-2262
PrimeSTAR® HS DNA Polymerase Kit	TaKaRa	R010A
In-Fusion® HD Cloning Kit	TaKaRa	639649
T4 DNA ligase kit	New England Biolabs®	M0202S
Penicillin- Streptomycin Amphotericin B (100X)	G BIOSCIENCES©	SBP7630
Blotting-Grade Blocker	Bio-Rad	1706404
Restriction endonuclease enzymes	New England Biolabs®	----
**Antibodies**		
Pierce™ Anti-c-Myc Agarose	Thermo Fisher Scientific	20168
Mouse monoclonal anti-Grl3p (5E9)	Turkewitz's lab	Bowman et al., 2005
Mouse monoclonal anti-Grt1p (4D11)	Turkewitz's lab	Turkewitz and Kelly, 1992
Purified Anti-GFP (mouse)	Biolegend	902601
Anti-c-myc antibody, Mouse monoclonal	Sigma Aldrich	M44439
Rat anti mouse- IgG-HRP for IP	Abcam	ab131368
Goat anti Rabbit-HRP	Bio-Rad	1706515
Goat anti mouse-HRP	Bio-Rad	1706516
Rabbit anti Grl (P40) for WB	Turkewitz's lab	Kumar et al., 2014
Rabbit Anti-GFP Antibody	Invitrogen	A11122
Anti-Rabbit-488	Invitrogen	A21206

Journal of Cell Science • Supplementary information

Anti-Mouse-Texas Red	Invitrogen	T-6390
Rabbit anti-Grl3 ab	Turkewitz's lab	Kumar et al., 2014
Rabbit anti Grl1 for IF	Turkewitz's lab	Kuppannan et al., 2022; Ota, 2018

Table S4. Primer used for the study

**Identifier**	**Primer name**	**Sequence (5'-3')**
**SKPX01**	KO-V-ATPAse-a1-5UTR_F	GGGAACAAAAGCTGGTTTCTTATCTCAGCTCATATTACTTTTTTTAAG
**SKPX02**	KO-V-ATPAse-a1-5UTR_R	CCGCCACCGCGGTGGTTTAATAATTATAGTTTAATTATGTGCTTAACTCG
**SKPX03**	KO-V-ATPAse-a1-3UTR_F	TACCGTCGACCTCGAATAAGCAAACAATCTGTTTTAATTTAATTTAATATAC
**SKPX04**	KO-V-ATPAse-a1-3UTR_R	CGGGCCCCCCCTCGAATCTTTTACACCTTCATCACCAATTTC
**SKP49**	V-ATPase-a1-RT-PCR_F	GGACTTGGGAAAGGAGATG
**SKP50**	V-ATPase-a1-RT-PCR_R	TTAGTTCCTGTTGTTTCGATGG
**SKP86**	V-ATPAse-a1-gene_F to amplify V-ATPase Gene to insert into p2XmNeon-Neo4 vector	ACCGCGGTGGCGGCCATGATTTTTTAACAAATTTTTAGAT
**SKP88**	V-ATPAse-a1-gene_R to amplify V-ATPase Gene to insert into p2XmNeon-Neo4 vector	AGTTCTAGAGCGGCCGTTGTTTTCATAATAAATTTGATGC
**SKP89**	V-ATPAse-a1-3UTR_F to amplify V-ATPase 3'UTR to insert into p2XmNeon-Neo4 vector	TACCGTCGACCTCGAATAAGCAAACAATCTGTT
**SKP90**	V-ATPAse-a1-3UTR_F to amplify V-ATPase 3'UTR to be inserted into p2XmNeon-Neo4 vector	CGGGCCCCCCCTCGAATCTTTTACACCTTCATCAC
**SKP 17**	M13_R	CAGGAAACAGCTATGACC
**SKP 18**	M13_F	TGTAAAACGACGGCCAGT
**SKP424**	CHX_Pst1_Infusion_F	agctgtagttagttCGCGAACTGAATCGGTCAG
**SKP425**	CHX_Xma1_Infusion_R	ATTCAGATCCCCCGGGGGCTGCATTTTTCCAGTAA
**SKP507**	V-ATPase-a2-Gene_F	ACCGCGGTGGCGGCCCTCACTTAAGATGAAAACCAGTGTTG
**SKP508**	V-ATPase-a2-Gene_R	AGTTCTAGAGCGGCCATATCTCTTTTCTACAGCAGCAACAG
**SKP509**	V-ATPase-a2-3UTR_F	TACCGTCGACCTCGACGAGACTAGAATTACTGACTG
**SKP510**	V-ATPase-a2-3UTR_R	CGGGCCCCCCCTCGATGGAAGTTTGCTATGAAATCAG
**SKP514**	VMA1-Gene_F	ACCGCGGTGGCGGCCCAGTCTCTACTCTTGCTATCGTAC
**SKP515**	VMA1-Gene_R	AGTTCTAGAGCGGCCACGGTCATTGATTTTTCTGAAAGC

Journal of Cell Science • Supplementary information

**SKP516**	VMA1-3UTR_F	TACCGTCGACCTCGAGCTACCCTATACTTATTTTGATATACCTCTG
**SKP517**	VMA1-3UTR_R	CGGGCCCCCCCTCGAACCTCCCTGACTTTAAATTTCTCTTC
**SKP538**	VMA8-Gene_F	ACCGCGGTGGCGGCCATATTGCAGGTGTTATGTTACC
**SKP539**	VMA8-Gene_R	AGTTCTAGAGCGGCCAACAACTATATCCTCATCAGCTTC
**SKP540**	VMA8-3UTR_F	TACCGTCGACCTCGATAGTGTTTGTTTGTTTATCTTCCTTG
**SKP541**	VMA8-3UTR_R	CGGGCCCCCCCTCGAATCCTTATTCATCAATAACTTTAACTCC
**SKP544**	VMA10-Gene_F	ACCGCGGTGGCGGCCCTAAAGATGAGCAATTCTAACGC
**SKP545**	VMA10-Gene_R	AGTTCTAGAGCGGCCCTTTTATTTCATTTTGCTGAAATCACC
**SKP546**	VMA10-Gene_F	TACCGTCGACCTCGATTTAATCAAGCAAGAGTGTAAAAGTTAG
**SKP547**	VMA10-Gene_R	CGGGCCCCCCCTCGAACCCCAGATTAAAGACTAAAAATAGG
**SKP51**	Btu1_F	ATGAGAGAAATCGTTCACATC
**SKP52**	Btu1_R	TGACCGAAAACGAAGTTATC
**SKP751**	mCherry_SpeI_F	GCTTTACAAAACTAGTTCCGGATCCATGGTTTCTAAAGGTGAAGAAG
**SKP750**	mCherry_SpeI_R	AGTTCGCTCAACTAGTTCACTCGAGGACGTCTTTATATAATTC

Table S5. Related to Fig. 1A and 1B.

	V-ATPase-a1	*V-ATPase-a2*	*V-ATPase-a3*	*V-ATPase-a4*	*V-ATPase-a5*	*V-ATPase-a6*	*GRL1*	*CTH3*	*GRT1*
Ll	2292.28	2609.72	3351.15	5160.37	15672.03	555.08	43105.33	5745.49	38275.89
Lm	935.84	1247.53	2862.96	3196.19	18404	793.1	33766.79	4076.88	34757.92
Lh	1741.41	1890.43	3511.25	3927.79	18109.9	626.72	39616.13	5084.34	39497.71
S0	5786.33	617.73	2724.14	2426.41	11200.11	585.55	60831.32	22459.57	59366.64
S3	4861.86	751.78	4545.06	2497.2	11395.55	399.11	59837.94	15102.49	56188.12
S6	1905.66	280.09	3912.14	2249.4	12040.2	404.57	57581.78	7791.38	54360.99
S9	1024.07	320.87	4979.53	2296.36	11878.59	302.68	37182.12	2813.96	31324.37
S12	637.41	343.25	4710.3	2374.51	12026.48	327.35	19880.7	1486.46	16125.46
S15	573.08	361.67	4678.5	2532.74	11859.41	324.39	13264.78	1032.11	11830.72
S24	566.7	351.87	4928.56	2508.4	13163.25	415.37	21608.8	1329.89	17224.27
C0	3522.38	229.24	5994.22	1234.04	11968.34	372.52	58140.54	10633.82	53931.26
C2	5474.64	938.02	1860.24	1273.98	6722.05	321.69	61360.03	19355.87	61027.69
C4	2863.16	541.57	5982.63	2222.15	2901.38	186.3	59882.28	8540.12	59896.27
C6	1003.76	210.02	3953.54	1832.52	2613.78	166.7	45554.15	2450.49	42057.23
C8	445.05	211.22	2207.98	1777.7	12253.19	256.25	31135.9	1330.9	27416.33
C10	482.03	235.43	2416.17	1650.73	16853.64	281.51	35770.87	1427.73	26775.68
C12	1540.56	363.85	2718.25	1770.68	15418.8	358.26	55371.76	5155.58	48651.35
C14	2427.09	364.32	3050.02	1752.83	12965.04	239.36	53852.77	7337.5	52204.2
C16	2737.26	220.4	3457.7	1873.66	10708.65	250.63	59377.63	7937.21	59717.24
C18	2839.2	197.52	3672.2	1768.47	11036.2	213.82	58319.46	8178.36	57425.27

Journal of Cell Science • Supplementary information
